# Mesenchymal-endothelial nexus in breast cancer spheroids induces vasculogenesis and local invasion in a CAM model

**DOI:** 10.1038/s42003-022-04236-5

**Published:** 2022-11-27

**Authors:** Aijun Li, Simone Muenst, Julius Hoffman, Laurent Starck, Melika Sarem, Andreas Fischer, Gregor Hutter, V. Prasad Shastri

**Affiliations:** 1 Institute for Macromolecular Chemistry, University of Freiburg, Freiburg, 79104 Germany; 2 Institute for Medical Genetics and Pathology, University Hospital Basel, Basel, 4031 Switzerland; 3 Institute for Clinical Chemistry, Göttingen University Hospital, 37075 Göttingen, Germany; 4 Division Vascular Signaling and Cancer, German Cancer Research Center, 69120 Heidelberg, Germany; 5 Brain Tumor Immunotherapy Lab, Department of Biomedicine, University of Basel, Basel, 4031 Switzerland; 6 Department of Neurosurgery, University Hospital Basel, Basel, 4031 Switzerland; 7 BIOSS - Centre for Biological Signalling Studies, University of Freiburg, Freiburg, 79104 Germany

**Keywords:** Breast cancer, Cancer models

## Abstract

Interplay between non-cancerous cells (immune, fibroblasts, mesenchymal stromal cells (MSC), and endothelial cells (EC)) has been identified as vital in driving tumor progression. As studying such interactions in vivo is challenging, ex vivo systems that can recapitulate in vivo scenarios can aid in unraveling the factors impacting tumorigenesis and metastasis. Using the synthetic tumor microenvironment mimics (STEMs)—a spheroid system composed of breast cancer cells (BCC) with defined human MSC and EC fractions, here we show that EC organization into vascular structures is BC phenotype dependent, and independent of ERα expression in epithelial cancer cells, and involves MSC-mediated Notch1 signaling. In a 3D-bioprinted model system to mimic local invasion, MDA STEMs collectively respond to serum gradient and form invading cell clusters. STEMs grown on chick chorioallantoic membrane undergo local invasion to form CAM tumors that can anastomose with host vasculature and bear the typical hallmarks of human BC and this process requires both EC and MSC. This study provides a framework for developing well-defined in vitro systems, including patient-derived xenografts that recapitulate in vivo events, to investigate heterotypic cell interactions in tumors, to identify factors promoting tumor metastasis-related events, and possibly drug screening in the context of personalized medicine.

## Abstract (web summary)

The interplay of mesenchymal and endothelial cells with breast cancer (BC) cells are investigated in a synthetic tumor microenvironment mimics (STEM) and CAM model to assess migration and metastasis.

## Introduction

In the past decade, cancer has surpassed cardiovascular diseases as the main cause of fatality in the developed world and remains one of the prominent challenges of 21st century medicine^1^. Breast cancer (BC) is the most prevalent cancer among women in western society and metastasis is the greatest contributor to cancer-related deaths^2^. Since the late 1990s, significant advances have been made in detecting tumors (tumor-associated and secreted biomarkers, circulating tumor cells (CTCs))^3,4^, and the treatment of tumors (targeted nanotherapeutics, checkpoint inhibitors, and immunotherapy)^5,6^, and these have contributed tremendously to the arsenal to fight cancer. However, there is emerging clinical evidence that metastasis of a tumor to a distant organ can occur several years before the primary tumor is even detected^7,8^. This brings to focus the importance of understanding the drivers of early tumor formation and the factors promoting local invasion that are critical to primary tumor growth and progression.

Solid epithelial tumors are a heterogeneous composition of different types of cells, including cancer cells, stromal or support cells (mesenchymal stromal cells (MSC), fibroblasts), and immune cells, and are characterized by the presence of unorthodox blood vessels originating from aberrant endothelial cell organization^9,10^. BC starts as a local disease, and how it acquires its metastatic capacity is not yet fully understood. It is postulated that metastatic capacity is acquired during tumorigenesis and involves distinct subpopulations of cells within the tumor that have undergone genetic alterations enabling their dissemination^11^. Moreover, the acquisition of stromal and immune cells by solid epithelial tumors is considered to be a pivotal step in the evolution of the tumor microenvironment (TME) and the progression of tumors^12^. Dynamic temporal changes to cellular composition of the stromal and immune compartments such as infiltration of cancer-associated fibroblasts and macrophages play a critical role in angiogenesis, tumor cell motility, and extravasation, and also aid in priming the pre-metastatic site^13^. Concordantly, a high macrophage content in the primary tumor correlates with poor patient prognosis^14^. These findings suggest that tumor heterogeneity is an important trait in the malignancy status^15,16^. To date, the focus has primarily been on elucidating the role of immune cells such as T-regs^17–19^, and their interaction with MSC in regulating the host-immune response to tumors and tumor progression^20–22^. Recent studies have highlighted the importance of the cross-talk between non-cancer cells in driving tumor growth and have brought to focus the importance of the soil in the Paget’s hypothesis.

To investigate heterotypic interactions between cells within the TME multicellular systems comprising of cancer cells, fibroblasts, and/or endothelial cells (EC) have been studied in hanging drop spheroids, microfluidic chip and polymer-gel scaffolds^23–27^. Many of these studies have employed transformed fibroblasts and/or human umbilical vein EC both of which are not physiologically consistent with tumor microenvironment. To address this and in order to recapitulate early cellular infiltration events in the tumor, we proposed a spheroid system composed of well-defined fraction of human tumor cells with human primary microvascular EC and human bone-marrow-derived MSC and termed it synthetic tumor microenvironment mimics (STEMs), and showed the STEMs can recapitulate many of the traits of lung adenocarcinoma microenvironment^28^. This system is ideal for studying the role of stroma in tumorigenesis as MSC is one of the sources for cancer-associated fibroblasts is marrow-derived^29^, and furthermore, MSC plays multiple roles in the TME including immunomodulation. Using the STEMs system, we had identified a role for MSC in sustaining an EC population in the hypoxic core of the spheroids. In this study, building on our earlier findings, we investigate the role of MSC-EC crosstalk in the cellular organization in spheroids derived from two BC cell lines MCF-7 and MDA-MB-231 and found that the organization of EC into vascular structures is governed by BCC phenotype and requires MSC. Furthermore, the migratory status of MSC is modulated by BCC phenotype and in an EC-dependent-manner. Remarkably, both MDA- and MCF STEMs acquire local invasive capacity on chick chorioallantoic membrane to form CAM tumors and this process requires both MSC and EC. This suggests an important role for MSC-EC crosstalk in collective tumor cell migration and progression of breast tumors into a more invasive phenotype and adds to the findings by Weinberg and others on a pro-metastatic role for MSC in breast cancer^20,21,30^.

## Results and discussion

### Mesenchymal-endothelial cell interaction drives BC epithelial organization

**Fig. 1:**
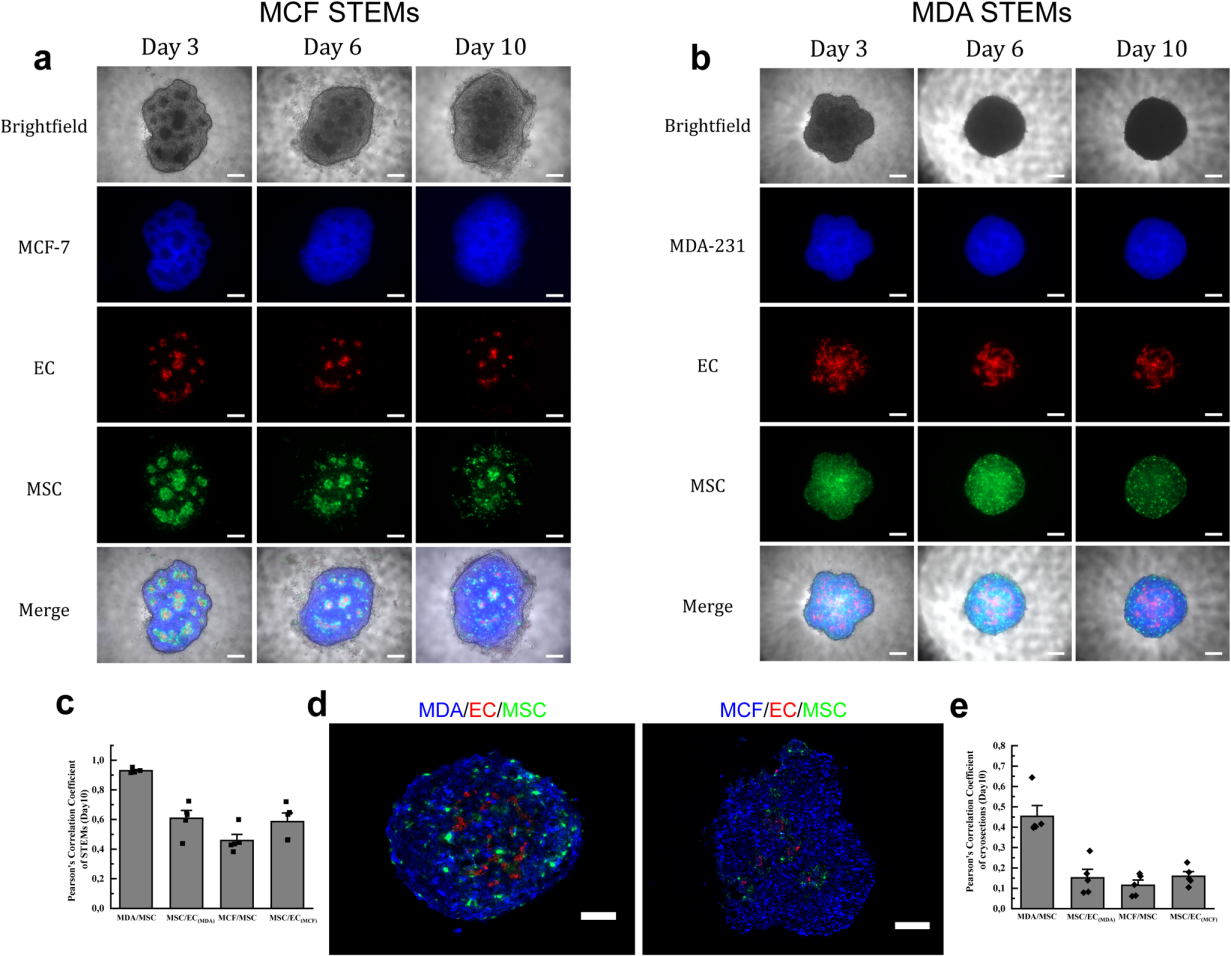
Organization of BCC in STEMs is determined by MSC-EC interactions. **a** Brightfield and fluorescence microscopy images (MCF-7/BFP, EC/tdTomato, and MSC/GFP) of MCF STEMs, scale bar: 200 µm. **b** Brightfield and fluorescence microscopy images (MDA-231/BFP, EC/tdTomato, and MSC/GFP) of MDA STEMs, scale bar: 200 µm. **c** Pearson correlation coefficients (PCCs) of MDA/MSC, MSC/EC_(MDA),_ MCF/MSC, MSC/EC_(MCF)_ of STEMs after 10 days, *n* = 5. **d** Fluorescence images of cryosections of MDA and MCF STEMs on day 10 showing the distinct differences in the distribution of MSC and the organization of EC in the two systems. Note the close association between MSC and BC in MDA STEMs, which is lacking in the MCF STEMs, scale bar of the left image: 100 µm, scale bar of the right image: 200 µm. **e** PCCs of MDA/MSC, MSC/EC_(MDA),_ MCF/MSC, MSC/EC_(MCF)_ of cryosections after 10 days, *n* = 5.

The mutual and dynamic communication between cancer cells and stromal cells may exert an influence on tumor progression, metastasis, and patient prognosis^31^. To elucidate the role of MSC-EC heterotypic interactions in the cellular organization of BC spheroids, we first determined if the phenotype of epithelial cells plays any role. We used the scaffold-free hanging drop system^28,32,33^ and exploited the STEMs spheroid platform which has been shown to recapitulate critical features of lung adenocarcinoma^28^. In the STEMs system, epithelial spheroids are formed in presence of human MSC and EC to recapitulate a stromal TME. Based on our past work and literature reports^28,34–36^, here we chose a ratio of 5:3:2 of BC cells (BCC)/EC/MSC and additionally used cells stably expressing fluorescent proteins (BC cells: blue fluorescence protein (BFP), MSC: green fluorescence protein (GFP), EC: tdTomato) to follow cellular organization using fluorescence microscope. Since epithelial-mesenchymal transformation is considered a pivotal point in tumor invasion and metastasis, we first aimed to compare the formation and cellular organization using MCF-7 (MCF) and MDA-MB-231 (MDA), two cell lines derived from cells isolated from pleural effusions in patients with metastatic BC^37^, with the former being human estrogen receptor alpha (ERα) positive with low metastatic potential and considered a good model of early-stage disease, and the latter being triple-negative, highly invasive, and a model for late-stage BC^38^. Following the initiation of the hanging drop, the global organization of cells within the STEMs was followed over a 10-day period (3, 6, and 10 days) qualitatively using fluorescence microscope (Fig. 1a, b). A few striking features were immediately evident. First, in the MCF STEMs the BC were organized around islands comprising of MSC and EC, whereas in the case of the MDA STEMs, a prominent organization of EC showing strong association with MSC was observed. This pattern of the organization was evident on day 3 and continued to endure through day 10. Over 10 days, further maturation of the STEMs was observed with MCF STEMs continuing to exhibit irregular shapes, while MDA STEMs became condensed and circular.

Interestingly, in both systems, MSC and EC appeared to reside in close proximity. In order to develop a quantitative measure of the cellular association, we calculated Pearson correlation coefficients (PCC), which is a bivariant statistical method to determine a linear correlation between two data sets^39^. We found that MSC-EC (green/red) show clear (positive PCC values) in both cell systems with PCC values ranging from 0.4 to 0.6 (Fig. 1c). The more intriguing observation was a divergence in MSC interaction with BC in the two systems, with MSC showing a strong association with MDA cells but not with MCF. As photons in the images of the spheroids can originate at various depths, cryosections were analyzed, and the PCC values and cellular co-organization confirmed the general observations in the 3D environment (Fig. 1d, e). To ascertain the role of MSC and EC in the cellular organization, spheroids were prepared with only BCC, BCC with either MSC or EC, and it was found that none of these systems yielded the cellular organization observed in the STEMs (Supplementary Fig. 1), unraveling that the presence of three-way heterotypic interactions between BCC-MSC-EC plays an important role for cellular organization within BC microenvironments.

**Fig. 2:**
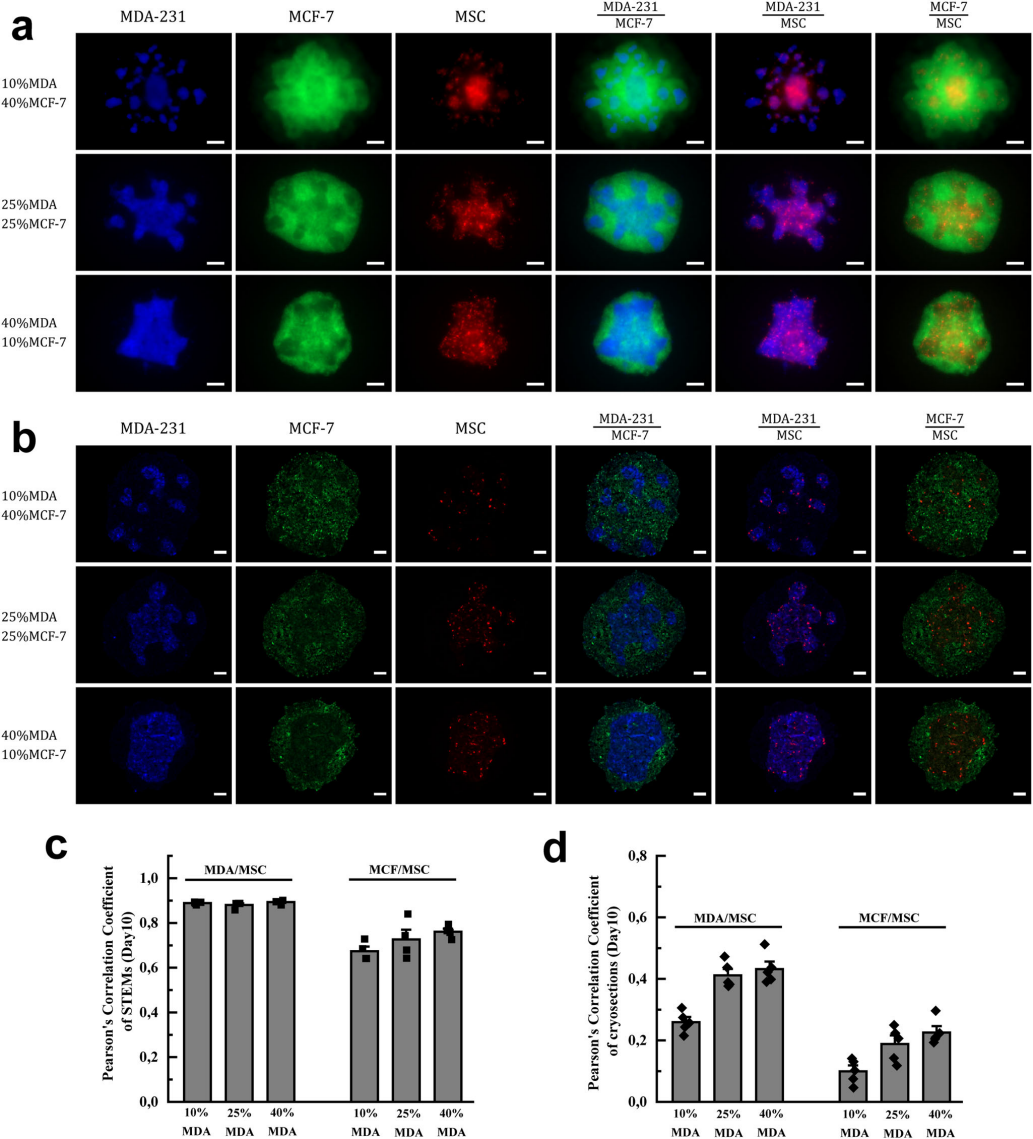
MDA promotes redistribution of MSC in MCF STEMs. **a** Fluorescence microscopy images of MCF STEMs bearing increasing fractions of metastatic cells (MDA-231/BFP, MCF-7/GFP, MSC/tdTomato, and EC (unlabeled), scale bar: 200 µm. The introduction of MDA drives the redistribution of MSC throughout the structure, providing evidence for associative tendencies between MSC-MDA. **b** Fluorescence microscopy images of cryosections of MCF-STEMs containing a different cellular fraction of MDA cells, scale bar: 100 µm. **c** PCCs of MDA/MSC and MCF/MSC in MCF-STEMs containing increasing fractions of MDA, *n* = 4. **d** PCCs of MDA/MSC and MCF/MSC in cryosections of MCF-STEMs containing increasing fractions of MDA, *n* = 5.

Since tumor aggressiveness shows a positive correlation with the emergence of immune and vascular compartments^40,41^, we aimed to ascertain if the observed cellular organization is driven by interactions between single cells or a morphological trait gained upon a large-scale organization. The importance of BCC-MSC-EC interaction in defining cellular organization is evident in the sorting behavior observed in the aggregates at 24 h preceding spheroid formation (Supplementary Fig. 2a, b). In MCF STEMs, EC/MSC clusters were encased by BCC forming the basis for the island organization observed later on, and likewise, in MDA STEMs, BCC organization around EC structures was evident, which is consistent with the organization of EC/MSC structures throughout the STEMs observed at later time points. PCC analysis provided strong evidence for the associative traits between the various cell populations in the two systems, and in particular, confirmed a strong association between MSC with BC in the MDA aggregates (Supplementary Fig. 2c). As MCF and MDA possess phenotypic differences, we investigated the role of the BCC phenotype in driving cellular organization by introducing MDA into MCF STEMs microenvironment. We found that increasing the MDA cellular fraction disrupted the island morphology observed in the MCF STEMs, and furthermore, drove a uniform re-distribution of MSC, which was evident in both 3D images and cryosections (Fig. 2a, b). Even in this mixed MDA-modified MCF STEMs environment, the associative tendency between MSC and MDA was confirmed by PCC analysis. Circumspect evidence for a role for BC phenotype in altering the interaction between MSC-BCC was also evident by the increased associative tendency between MSC/MCF in MDA-modified MCF STEMs in comparison to MCF STEMs (Fig. 2c, d). These are notable findings as they prove the interaction between BCC and stromal cells in the cellular organization within tumors, and allude to possible BCC-related changes in the MSC phenotype as discussed below.

### BCC-MSC-EC crosstalk drives vasculogenesis

**Fig. 3:**
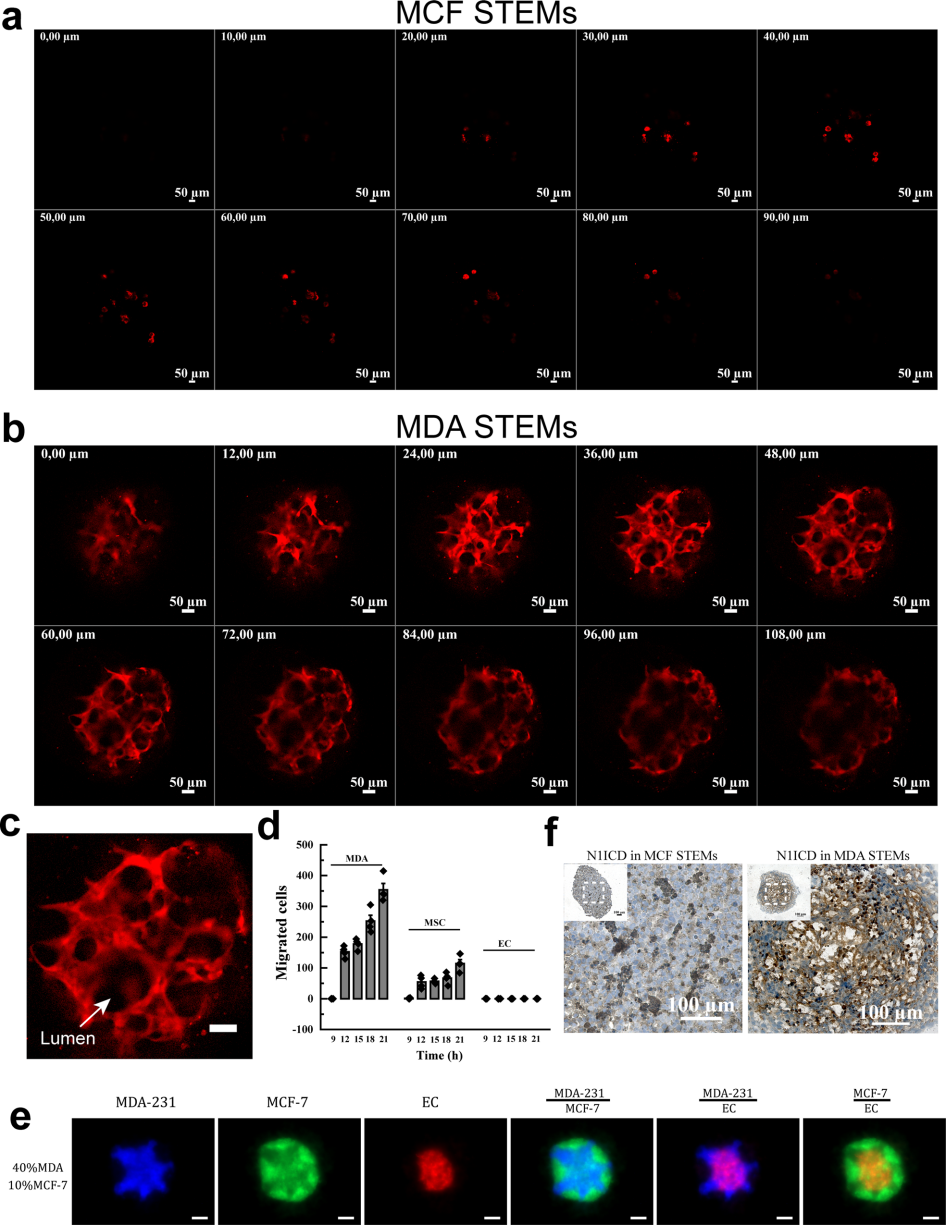
MDA STEMs promote vasculogenesis through MSC-activated Notch. **a** Confocal Z-stacks (thickness: 90 µm, 10 µm/slice) showing no organization of EC (tdTomato fluorescence) in the MCF STEMs. **b** Confocal Z-stacks (thickness: 108 µm, 12 µm/slice) showing organization of EC (tdTomato fluorescence) in the MDA STEMs. MDA STEMs environment uniquely promotes the organization of EC into vascular structures via vasculogenesis. **c** An exemplar image from confocal Z-stacks with high magnification, EC/tdTomato organization shows clear lumen structure (white arrow) in the MDA STEMs, scale bar: 50 µm. **d** Quantification of cell migration over time from MDA STEMs in transwell chambers, *n* = 4. **e** Fluorescence microscopy images of MCF STEMs bearing 40% MDA cells (MDA-231/BFP, MCF-7/GFP, EC/tdTomato, and MSC (unlabeled)), The images suggest MDA promotes reorganization of EC in MCF-STEMs, scale bar: 200 µm. **f** Immunohistochemical staining for N1ICD (activated Notch) in MCF and MDA STEMs cryosections. The strong staining (brown color) of N1ICD was clearly evident throughout MDA STEMs, while MCF STEMs did not show staining. The main images are high magnification of the dashed box area.

It is well known that tumors need to recruit new blood vessels to support their ever-increasing metabolic demand^42,43^, and that tumor angiogenesis is mandatory for metastasis^44,45^. Over the years, in addition to sprouting angiogenesis, other mechanisms for the formation of tumor vasculature have been proposed, including intussusceptive angiogenesis, vessel co-option, and vasculogenic mimicry^43^. Mural cells play an important role in the stabilization of such vascular structures^46,47^. Jain and co-workers have shown that MSC can function as mural support cells and promote the stabilization of vascular structures^48^. In spheroids containing fibroblasts and HUVEC^23,24^, outgrowth of HUVEC was closely associated with fibroblasts, and the formation of islands of HUVEC was observed in the core of the spheroid in close association with fibroblast as previously reported by us^28^. In a counter intuitive finding, here, confocal microscopy of the STEMs revealed that EC organized in vascular structures but in BC cell phenotype dependent manner. While EC within MDA STEMs had organized into vascular structures no such organization was evident in MCF STEMs (Fig. 3a, b and Supplementary Movies 1, 2). Furthermore, these vascular structures comprised of lumens (Fig. 3c) and stained positive for the apical marker podocalyxin (PODXL)^49^ (Supplementary Fig. 3). However, in absence of MSC, EC within the MDA environment did not form vascular structures (Supplementary Fig. 1b and Supplementary Movie 3). Furthermore, merged confocal images revealed a close association between MSC and EC indicating that juxtacrine interaction may play a role in the vascular structure formation (Supplementary Fig. 4). This finding is highly relevant as it identifies a clear role for BC phenotype in driving vasculogenesis. Since, the regrowth of tumors following irradiation has been postulated to involve vasculogenesis initiated by cross-talk between endothelial progenitor cells and cancer cells in the bone marrow^50^, and intussusceptive angiogenesis, which has been shown to occur in tumors^51^, is thought to occur after vasculogenesis and plays a role in the expansion of capillary plexus, our findings further strengthen the case for alternative mechanisms for nascent tumors to acquire vasculature involving BCC-MSC-EC signaling nexus.

### EC-BCC crosstalk modulates MSC migratory phenotype in BC STEMs microenvironment

In absence of any external vasculature the cues for vasculogenesis have to be endogenous in origin. EC migration constitutes an important step in capillary expansion by sprouting angiogenesis^52^. While the behavior of the BCC was consistent with what has been reported in the literature i.e., MDA undergoes migration, and MCF do not, unexpectedly, even though, MSC and EC show migratory behavior in 2D culture (Supplementary Fig. 5a) no out-migration of EC was observed from either system. Even more remarkably, while MSC migrated out of MDA STEMs, MSC migration was completely abrogated in MCF STEMs (Fig. 3d and Supplementary Fig. 5b). This suggests a possibly alternative mechanism for the organization of EC into vessel-like structures. Since a mixed culture media was employed and cells were transfected to express fluorescent proteins, we carried out migration studies in their respective culture media and the mixed media and found neither culture media nor transfection impacted the migration of cells thus eliminating these variables as the cause of our observations (Supplementary Fig. 5c–e). In addition, EC organization in MDA and MCF STEMs showed no discernible differences irrespective of whether they contained un-transfected or fluorescence protein transfected BCC/MSC confirming that fluorescent protein expressing in the cells had no impact on our observations (Supplementary Fig. 5f, g). Given that primary MSC isolated from human bone-marrow are heterogeneous, MDA and MCF STEMs were prepared using two additional donors and it was found that the cellular organization within these spheroids was not impacted by the donor (Supplementary Fig. 5h–k).

As EC-MSC show associative tendencies in both STEMs, we then probed the role and fate of MSC and found that the non-migratory status of EC was independent of the presence of MSC. However, when EC were removed from the MCF STEMs, MSC recovered their migratory phenotype (Supplementary Fig. 5l). The migratory phenotype in MSC could also be rescued by introducing MDA into MCF STEMs (Supplementary Fig. 5m). Furthermore, introduction of MDA into MCF STEMs led to the progressive organization of EC (Fig. 3e and Supplementary Fig. 6a, b, and Supplementary Movie 4). Merged confocal images (Supplementary Fig. 6c, d) showed the close association between MSC and EC. In sum, these findings provide strong evidence for a role of BCC phenotype in modulating MSC-EC interaction and further confirming the presence of a 3-way signaling paradigm between BCC-MSC-EC in driving MSC redistribution and EC organization. While, trans-differentiation of MSC into an endothelial-like phenotype, the so-called mesenchymal-endothelial transition, which has been shown to play a role in cardiac neovascularization^53^ could be a distinct possibility, this can be ruled out based on the organization of the other cell populations with respect to EC in STEMs (Fig. 3b, Supplementary Fig. 4, and Supplementary Movie 1).

### MSC-activates Notch signaling in EC in BCC phenotype dependent manner

Notch is a transmembrane receptor that mediates cell-cell communication, orchestrates the Notch signaling pathway, and plays an important role in cellular patterning and vasculogenesis during development and cancer growth^54–56^. Several functional studies in tumor models have shown that proliferation of EC and growth of the vascular network are controlled by Notch signaling^57^ with activation of Notch promoting EC quiescence via miR-218^58^, and regulating EC proliferation^59^. Activated Notch-1 receptor (N1ICD, Notch-1 intracellular domain) is found in EC in human carcinoma, and it has been shown that sustained vascular Notch signaling facilitates metastasis^60^. We carried out immunohistochemistry against N1ICD on cryo-sections of STEMs and found that while MDA STEMs showed intense staining that corresponded well with vascular structures, no detectable N1ICD staining was observed in MCF STEMs (Fig. 3f). Furthermore, MDA-EC spheroids was negative for N1ICD confirming that MSC are essential for Notch-1 signaling in MDA-STEMs (Supplementary Fig. 7a). Since Notch signaling requires cell-cell contact^61^ and has been shown to be proportional to cell contact area^62^, we used N-[(3,5-Difluorophenyl)acetyl]-L-alanyl-2-phenyl glycine-1,1-dimethyl ethyl ester (DAPT), a γ-secretase inhibitor that indirectly inhibits Notch signaling^63,64^ to see if this would impact the organization of MDA and MCF STEMs. While lower concentrations (10 µM, 25 µM) of DAPT have no discernible effect on MDA and MCF STEMs, exposure to 50 µM of DAPT led to fragmentation and blebbing in MDA STEMs and not in MCF STEMs (Supplementary Fig. 7b). This loss in cell-cell contact may account for the observed changes in the MDA-STEMs. Furthermore, MDA-EC and MCF-EC spheroids, which lack Notch-1 activation, were also treated by DAPT (Supplementary Fig. 8), it turns out that no discernible changes were observed even under the concentration of 50 µM. These observations taken in sum point to a role for MSC in activating EC-Notch signaling in STEMs which needs further investigation.

### EC organization into vascular structures is independent of ERα expression in epithelial cells

**Fig. 4:**
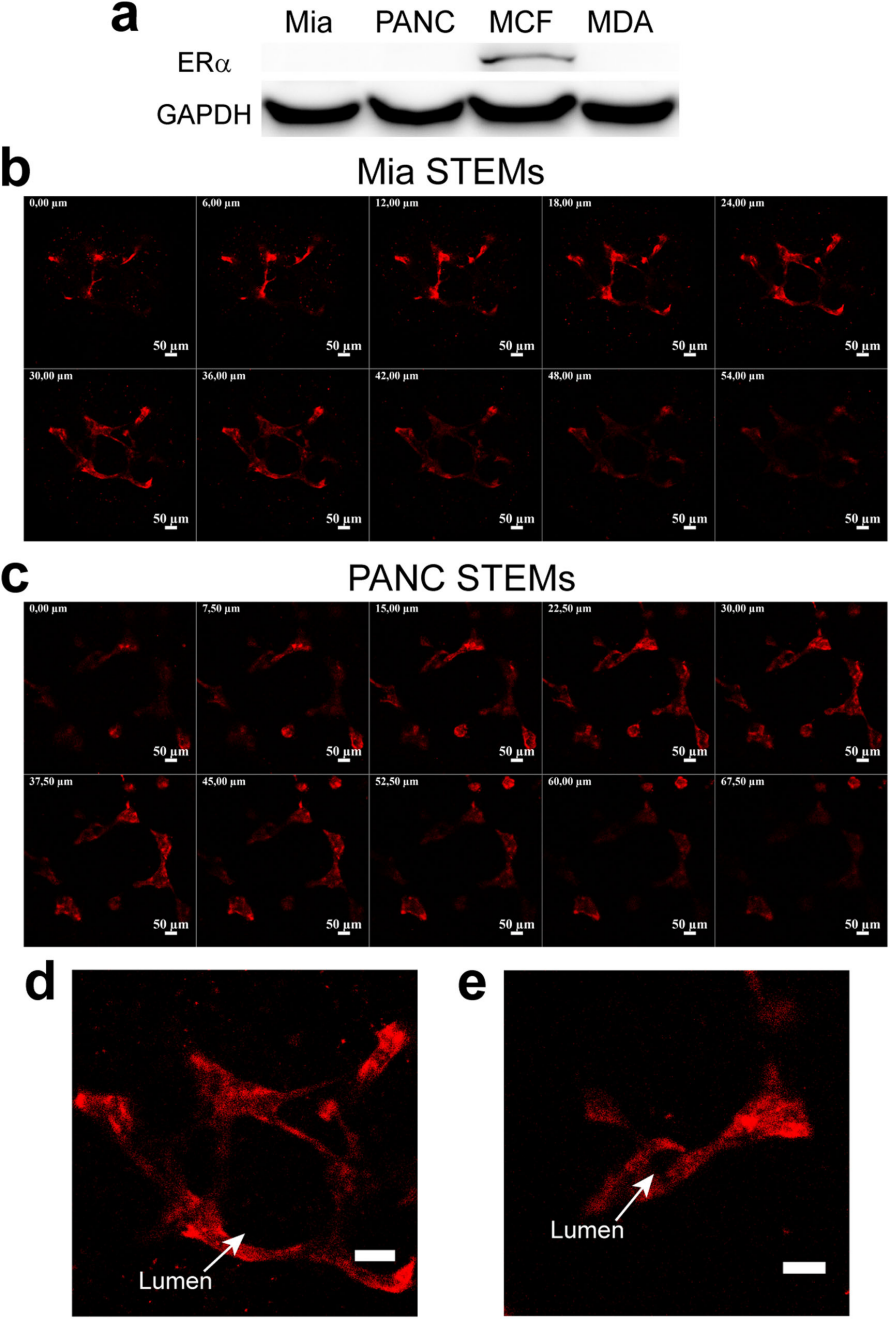
ERα-negative pancreatic cancer cell lines Mia-PaCa-2 and PANC-1 promote EC organization. **a** Western blot analysis of total cell protein lysate from Mia Paca-2, PANC-1, MCF-7, and MDA-MB-231 using antibody D-12 revealed a 66 kDa band confirming the expression ERα status in the cells. GAPDH was used as a loading control. **b** Confocal Z-stacks (thickness: 54 µm, 6 µm/slice) showing organization of EC (tdTomato fluorescence) in the Mia STEMs. **c** Confocal Z-stacks (thickness: 67.5 µm, 7.5 µm/slice) showing organization of EC (tdTomato fluorescence) in the PANC STEMs. **d** An exemplar image from confocal Z-stacks with high magnification, EC/tdTomato organization shows clear lumen structure (white arrow) in the Mia STEMs, scale bar: 50 µm. **e** An exemplar image from confocal Z-stacks with high magnification, EC/tdTomato organization shows clear lumen structure (white arrow) in the PANC STEMs, scale bar: 50 µm.

Notch stimulation has been associated with breast cancer cell motility^65^, and there is emerging evidence that mechanical forces play an important role in the activation of the Notch signaling pathway^62,66^. Since we had observed massive Notch activation in MDA STEMs, and since MDA cells are invasive and lack expression of ERα- receptor, we questioned if these attributes could be a prerequisite for driving MSC redistribution and EC organization in tumor environments. To test this hypothesis, we used two pancreatic cancer cell lines, Mia PaCa-2 and PANC-1, both of which are capable of migrating and metastasizing and lack expression of ERα- receptor^67^. We first verified the expression of ERα in MCF and the lack of ERα in MDA, Mia PaCa-2, and PANC-1 using western blot (Fig. 4a), and prepared STEMs of the pancreatic cancer cell lines with EC and MSC. We found that MSC showed distribution across the entire structure, close association with cancer cells (positive PCC values (Supplementary Fig. 9a), and more importantly, there was evidence for the organization of EC into vascular structures in both systems, with Mia PaCa-2 promoting structures akin to those observed in MDA STEMs, and PANC-1 promoting capillary-like elongated EC throughout the STEMs (Fig. 4b–e and Supplementary Fig. 9b, c, and Supplementary Movies 5, 6). Similarly, merged confocal images (Supplementary Figs. 10 and 11) also showed the strong association between MSC and EC.

To elucidate if the absence of ERα expression and migratory phenotype are sufficient to promote EC organization, we formed STEMs using MCF-10A, a non-tumorigenic mammary epithelial cell line, that can undergo migration but lacks ERα^68^, and found that while EC exhibit organization they lack well-defined large vascular structures observed in the ERα negative metastatic cancer cell STEMs (Supplementary Movie 7). This implies that a metastatic cancer cell phenotype in conjunction with the loss of ERα expression might represent a synergistic environment promoting vasculogenesis. To further examine our hypothesis, another highly metastatic, ERα negative BC cell line BT549^69^ was employed to generate spheroids. After six days the organization of EC into vascular structures was evident in this system (Supplementary Fig. 12a, b) with MSC closely associated with EC further confirming our findings (Supplementary Fig. 12c, d). Since, over-expression of ERα in human endometrial cancer line Ishikawa has been shown to limit tumor growth, inhibit vascularization, and reduce expression of α_v_β_3_ integrin which is necessary for angiogenesis^70^, and furthermore, in MDA, the reintroduction of ERα inhibited their metastatic potential^71^ and growth^72^, we inquired if introducing stable expression of ERα in MDA-MB-231 can abolish EC organization in the wild type MDA STEMs. STEMs were prepared using MDA transduced to stably express ERα (Supplementary Fig. 13a) and they revealed no changes to EC organization and MSC distribution compared to wild type MDA STEMs (Supplementary Fig. 13b–e), suggesting the inhibition effect of ERα expression was not sufficient to alter the cues in STEMs environment that promote the vascular organization. This implies that a metastatic cancer phenotype might offer other cues that induce vasculogenesis. This reasoning is confirmed by the observation that interfering with ERα signaling in MCF STEMs using Naloxone, a known antagonist of ER^73^ did not impact EC organization and cellular organization in general (Supplementary Fig. 13f).

### STEMs exhibit coordinated and collective migration in a 3D-bioprinted model system

**Fig. 5:**
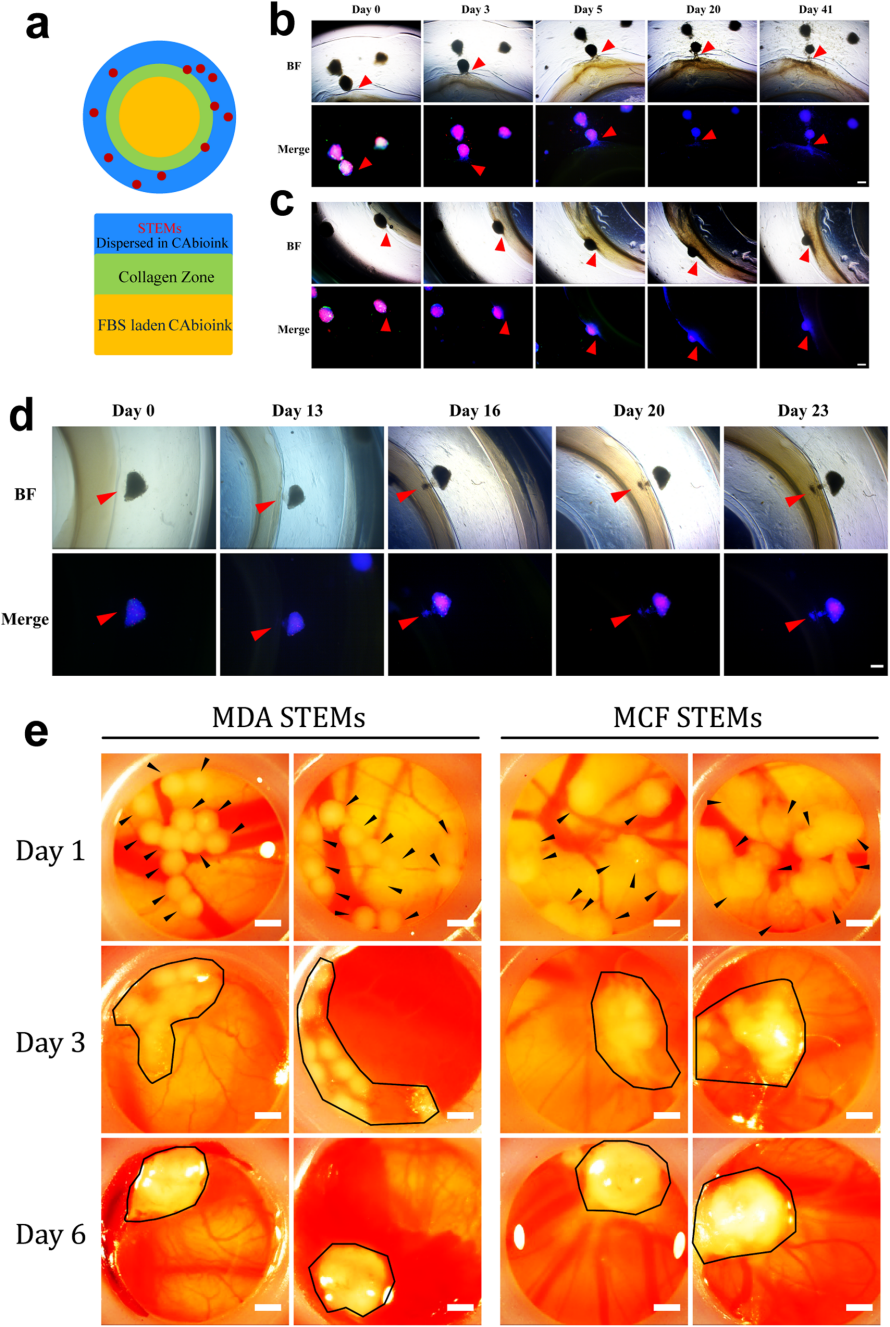
MDA STEMs exhibit collective migration in a 3D-Bioprinted model system and undergo local invasion in CAM environment. **a** Schematic on 3D-Bioprinted invasion assay system comprising of a construct of two conjoined concentric rings presenting the STEMs in a CA bioink, a collagen zone to mimic basement membrane, encompassing a center laden with the fetal bovine serum to establish a radial gradient of soluble signals. **b** Brightfield (BF) and fluorescence microscopy images of MDA STEMs collectively tugging (collective traction) at the collagen barrier and deforming it and over days disseminating BCC into the collagen. Red arrows point to the area of collective traction. **c** BF and fluorescence microscopy images of MDA STEMs invading (pushing into) the collagen and inducing a noticeable strain discernible by the depression (change in curvature) of the collagen, followed by dissemination of BCC into the collagen clearly noticeable by the blue-colored regions of BCC expressing BFP. Red arrows point to the area of invasion. **d** Image sequences showing the process of formation of a cell cluster (tumor metastatic unit) from a MDA STEMs and the invasion of the cell cluster (day 16) into the collagen zone and the formation of a subsequent daughter cell cluster in the collagen (day 20, 23), providing insights into the potential formation of CTC clusters. Red arrows point to the cell cluster. **e** Exemplar optical micrographs showing the process of local invasion and coalescence of STEMs inoculated on chicken CAM into CAM tumors. Day 1: Black arrows point to STEMs on the CAM membrane, Day 3: Areas bounded by a black line identify STEMs that are undergoing coalescence, Day 6: Areas bounded by the black line identify CAM tumors. Scale bar: 500 µm.

There is emerging evidence that distant metastasis can occur earlier than detection of the primary tumor^7,8^. Furthermore, CTCs have been found to exist as clusters (CTC-clusters), with tissue-derived macrophages and immune cells accompanying epithelial cells and acting as chaperons^74,75^ Compared to single CTCs, CTC-clusters have been shown to have 25–40-fold higher metastatic potential^76^. Invasion assay using Matrigel revealed that while no invasion of any cell population was observed from MCF STEMs (Supplementary Fig. 14), in MDA STEMs, both MSC and MDA cells invaded the Matrigel, with MSC preceding MDA cells (Supplementary Fig. 15). This provides further circumspect evidence for associative/cooperative interactions between MSC and metastatic MDA BC phenotype. This prompted us to investigate if MDA STEMs could recapitulate metastatic events associated with the primary tumor. To answer this question, we conceptualized an invasion experiment where STEMs are introduced into a biologically inert environment that they are incapable of remodeling and forced to respond to a chemokine gradient which is presented towards the STEMs across a biological barrier (Fig. 5a). To realize this experiment paradigm, we employed 3D bioprinting, a powerful emerging tool in studying tissue morphogenesis and tumor-associated events in well-defined environments^77^. We printed a construct comprising of two conjoined concentric rings, encompassing a center laden with the fetal bovine serum to establish a radial gradient of chemokines. The inner concentric ring was printed using collagen, which mimics the biological barriers that tumors (or BCC) have to remodel and traverse, and the MDA STEMs (10-15 per ring) were printed in the outer concentric ring using carboxylated agarose (CA) hydrogel-based bioink^78^. CA bioink was chosen as it is biologically inert, can support cell growth but cannot be remodeled by the cells^77^, thus ensuring that STEMs remain intact over the duration of the experiment, and their collective behavior elucidated. We observed that MDA STEMs are capable of responding collectively to serum-derived soluble signals. In some instances, we were able to capture MDA STEMs exerting collective strain (traction) on the collagen barrier by day 3, progressively pulling the collagen matrix and disseminating BCC into the matrix clearly by day 20 and subsequently withdrawing from the barrier by day 41 (Fig. 5b). In contrast, MCF STEMs did not show any collective behaviors (Supplementary Fig. 16).

In another scenario, we could observe MDA STEMs pushing at the collagen barrier starting at day 0 and collectively inserting themselves into the collagen barrier over the next several days. Over the next several weeks, they disseminated BCC into the collagen barrier and were securely anchored to the collagen barrier by 6 weeks (Fig. 5c). An even more intriguing finding occurred in MDA STEMs in close proximity to the collagen barrier, where after two weeks a small cluster of cells comprising primarily of MDA and few EC and MSC, started to pinch-off as a unit and migrate against the serum gradient and towards the collagen zone, finally penetrating the collagen by day 16 (Fig. 5d). Such observations, although infrequent, occurred in several STEMs (Supplementary Fig. 17). This heterogeneous caravan of cells, which we have termed—tumor metastatic unit—continued to undergo dissemination in the collagen over the next seven days. Since CTCs have been shown to travel the blood circulation as clusters^74,75^, this observation provides a potential glimpse into early events in the primary tumor that might precede local and distant metastasis. Taken together, these observations allude to an innate capacity for collective behavior in MDA STEMs.

### STEMs undergo local invasion in CAM assay to form tumors

Since the above observations occurred in absence of endocrine signaling and active circulation, we embarked on investigating the fate of STEMs in a living system. We chose the chick chorioallantoic membrane (CAM) assay, as this in (ex) ovo system possesses several important attributes, namely, a naturally immune-deficient host, and an actively remodeling vasculature that can bring inflammatory cues that are necessary for the evolution of an authentic TME. The CAM was pioneered by Murphy and Rous in 1912^79^ and then further developed by Folkman and co-workers in the early 1970s^80^. CAM has since been extensively used as a model to study angiogenesis and oncogenesis in neoplastic tissues^81^, and more recently, to study the motility and invasion behavior of ovarian cancer cell lines^82^. The *ex ovo* approach, where the embryo is removed from the egg and placed in a petri dish was chosen, as this allows for easy observation. Embryos were inoculated with MDA or MCF STEMs (10-12 STEMs/embryo) and their fate was assessed over a 6-day period. We made the surprising finding that both MDA and MCF STEMs coalesced through a collective invasion process we have termed local invasion and formed a single large structure referred to henceforth as CAM tumors (Fig. 5e). However, the propensity for such local invasion was greater in MDA STEMs with 75% (9 of 12) of the embryos yielding such a structure, while only 30% (4/13) of MCF STEMs yielding such structures.

### CAM tumors possess typical histopathological characteristics of human breast cancer

**Fig. 6:**
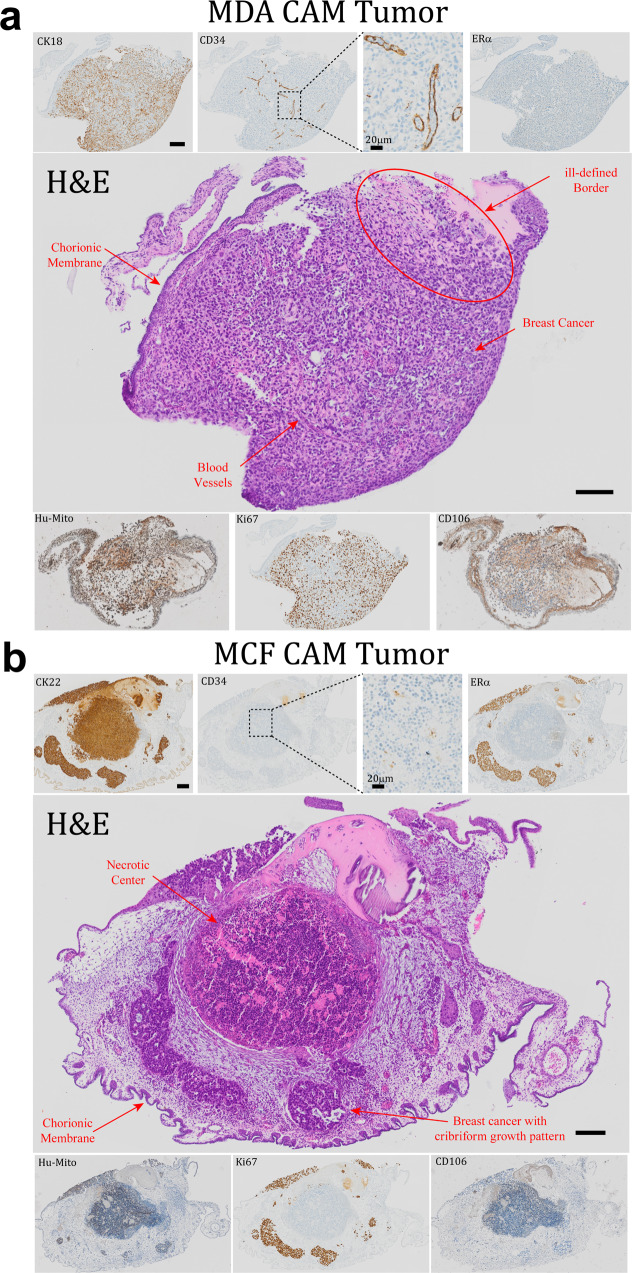
CAM tumors possess typical characteristics of human breast cancers. **a** MDA CAM tumor: H&E staining revealing a distinct small CAM tumor with clearly visible blood vessels and ill-defined borders, morphologically reminiscent of a small breast cancer lesion. Cancer cells within the CAM tumors were positive for CK18 and human mitochondria (Hu-Mito) and negative for human ERα. Anti-human CD34 staining revealed blood vessels lined by human EC and populated with nucleated avian erythrocytes suggesting functional anastomoses with the chicken embryo circulation. Faint staining for CD106 in the blood vessels points to EC that have been activated by factors secreted by the BCC. Lack of nuclear p63 (Supplementary Fig. 18a) and >40% positivity for Ki67 positivity is indicative of a more aggressive or metaplastic subtype of BC and corresponds to a grade G3. Scale bar: 100 µm. **b** MCF CAM tumor: H&E staining revealing cribriform structures in CAM tumor strongly resembling human breast cancer glands of a well or moderately differentiated breast cancer, with a necrotic center consistent with the absence of vasculature (negative for CD34). Positivity for CK22, Hu-Mito, ERα, and PR; and negative staining for Her2 (Supplementary Fig. 18c) confirm that the CAM tumors stem from human BCC and more specifically from hormone receptor (luminal) MCF-7 cells. Negative staining for CD106 confirms the avascular nature and the absence of stromal cell characteristics in MCF CAM tumor. The absence of myoepithelial cells (p63 -ve, Calponin -ve (Supplementary Fig. 18c)) and widespread positivity for Ki-67 (>90%) are indicative of an invasive BC phenotype corresponding to a grade of G2. Scale bar: 100 µm.

To ascertain if the coalesced structure was just a phenomenon of aggregation or a more complex cellular organization event driven by endogenous cues coming from the living CAM environment, CAM tumors were stained using a clinically relevant panel of markers for human breast cancer^83–85^ (Supplementary Table 1) and assessed by a board-certified pathologist specialized in breast pathology, and given a grade based on the morphological characteristics (Fig. 6a, b).

#### Histopathology of MDA CAM tumors

Histologically, coalesced MDA-STEMs formed distinct small CAM tumors with ill-defined borders, morphologically reminiscent of a small invasive ductal breast cancer focus with solid growth, distinct cellular atypia, and discernible mitotic activity, corresponding to poorly differentiated breast cancer (G3). The cancer cells were negative for human ERα, human progesterone receptor (PR), and Her2 (ERBB2)—human epidermal growth factor receptor (Supplementary Fig. 18a), proving that they stem from the triple-negative MDA BCC. Expression of cytokeratin-18 (CK18) and positive staining with Hu-Mito confirmed that the cells are human epithelial (cancer) cells. More importantly, staining for CD34, an EC marker, revealed the presence of blood vessels within the CAM tumors, proving that the MDA STEMs are able to induce vascular structures reminiscent of human BC vasculature. Since the considerably sized CAM tumors did not show any ischemic changes, the newly formed blood vessels are indeed open and functional, which was confirmed by the presence of nucleated avian erythrocytes in the vessels. CD106/ vascular cell adhesion molecule 1 (VCAM-1) is a protein that is expressed only by activated EC and is also a marker for the mesenchymal phenotype. Positive staining for VCAM-1 confirmed that a large subgroup of cells in this CAM tumor have a MSC phenotype. More interestingly, a detectable, albeit faint staining for VCAM-1 in the newly formed blood vessels was evident, suggesting that these EC had been activated by factors secreted by the cancer cells, providing evidence for the evolution of tumor-specific vasculature. The presence of very few p63 positive cells (Supplementary Fig. 18a) and Ki-67 positivity of about 40% suggests that the CAM tumor is an aggressive, metaplastic subtype of BC which is actively proliferating.

#### Histopathology of MCF CAM tumors

Histologically, coalesced MCF STEMs formed distinct cribriform structures in the chicken stroma, strongly resembling human breast cancer glands of a moderately differentiated invasive ductal breast cancer (G2). The center was almost completely necrotic and cancer had spread to the chicken chorionic membrane, imitating a pleural or peritoneal carcinosis. Since MCF STEMs did not show the cribriform organization of epithelial cells in vitro (Supplementary Fig. 18b), the formation of these structures seemed to have occurred *de novo* as a consequence of the processes of coalescence of the STEMs mimicking the tumorigenesis process occurring in vivo. The cancer cells showed membranous staining for cytokeratin’s (CK22) and strong nuclear staining for human ERα and PR, while being negative for Her2 (Supplementary Fig. 18c), and furthermore positive for Hu-Mito, confirming a luminal phenotype consistent with MCF and the human origins of CAM tumors. Importantly, in contrast to the MDA, MCF CAM tumors lacked vascular structures (CD34 negative), which corresponds to the observed necrosis of the CAM tumor. A negative staining for p63 and Calponin (Supplementary Fig. 18c), and a high Ki-67 positivity of around 90% in the CAM tumor which was higher than MDA, points to a transformation of the low-metastatic MCF-7 into a more aggressive phenotype in the CAM environment. On the other hand, immunohistochemistry against N1ICD on both MDA and MCF CAM tumor was performed and found that MDA CAM tumor showed intense staining that corresponded well with blood vessels, no detectable N1ICD staining was observed in MCF CAM tumor (Supplementary Fig. 19).

### CAM tumor formation requires MSC-EC crosstalk

Since we were able to prove a role for MSC-EC interaction for the cellular organization and vasculogenesis in the STEMs, and also have confirmed that the CAM tumors bear resemblance to human BC, we next investigated if the formation of CAM tumors requires EC and/or MSC. CAMs were inoculated with spheroids of MDA and MCF alone, as well as spheroids of MDA and MCF composed of either MSC or EC, and their fate was followed over a 6-day period. We made the observation that none of the spheroids were capable of forming CAM tumors and in fact, all the spheroids had undergone autolysis by day 3 (Supplementary Fig. 20). This finding provides the most compelling evidence for the presence of a 3-way signaling paradigm between BCC-MSC-EC in tumors and its essential role in collective migration of cancer cells and BC metastasis.

## Conclusion

In this study, using STEMs, an engineered tumor microenvironment with defined MSC and EC composition, a clear role for three-way signaling paradigm between BCC-MSC-EC in driving cellular organization and *de novo* organization of EC into vascular structures has been identified. The formation of vascular structures has been shown to involve activation of Notch signaling in EC by MSC and this, in turn, is impacted by the phenotype of BCC. The loss of ERα expression in epithelial cells appears to be part of a larger phenotype transformation paradigm and could represent an important step in tumor-angiogenesis. By combining STEMs with 3D-bioprinting, events associated with tumorigenesis including coordinated collective migration in response to a chemokine gradient, dissemination of BCC across a biological barrier, and the formation of a caravan of cells—tumor metastatic unit—was observed. The implication of this observation was verified in the CAM assay, where STEMs underwent local invasion to give rise to CAM tumors that possessed the typical hallmarks of invasive human BC. This study provides a framework for developing well-defined in vitro systems, including patient-derived xenografts that recapitulate in vivo events, to investigate heterotypic cell interactions in tumors, to identify factors that promote tumor metastasis-related events, and possibly drug screening in the context of personalized medicine.

## Methods

### Cell culture and reagents

The human breast cancer cell lines (MDA-MB-231 and MCF-7), the human pancreatic cancer cell lines (Mia Paca-2 and PNAC-1), MCF-10A, and HEK293 cells were provided by the toolbox of BIOSS (Center for Biological Signaling Studies, University of Freiburg) and were genotyped and verified by Labor für DNA Analytik (Freiburg, Germany). BT549 cells were kindly provided by DKFZ (Heidelberg, Germany) and were authenticated using Multiplex Cell Authentication by Multiplexion^86^. The SNP profiles matched known profiles or were unique. Breast and pancreatic cancer cell lines and HEK293 were cultured in Dulbecco’s Modified Eagle’s Medium (DMEM) (Gibco, Thermo Fisher) supplemented with 10% fetal bovine serum (FBS) (Thermo Fisher), and 1% penicillin-streptomycin (PAN Biotech, Germany). BT549 cells were cultured in RPMI1640 (Gibco, #A1049101) with 10% FBS, 1% penicillin-streptomycin, 2- mM glutamine (Gibco, #25030-024) and 10 μg/ml insulin (Sigma, #91077C). MCF-10A was cultured in DMEM/F12 (Invitrogen, #11330-032) with 5% horse serum (Invitrogen, #16050-122), 20 ng/ml EGF (R&D Systems, #236-EG-200), 0.5 mg/ml hydrocortisone (Sigma, #H-0888), 100 ng/ml cholera toxin (Sigma, #C-8052), 10 μg/ml insulin (Sigma, #I-1882) and 1% penicillin-streptomycin. Human pulmonary microvascular endothelial cells (HPMEC) were purchased from PromoCell (Heidelberg, Germany) and were propagated in the Growth Medium MV (Ready-to-use) (PromoCell: C-22020). Human marrow-derived mesenchymal stromal cells (MSC) were kindly provided by Dr. Andrea Barbero and were obtained from patients under consent per the regulations of the local ethical committee (University Hospital Basel; Ref No: 78/07). MSC were sub-cultured in alpha-MEM (Gibco) containing 10% FBS, 1% penicillin-streptomycin, and 5 ng/mL fibroblast growth factor-2 (R&D Systems). HPMEC and MSC were used up to passage five. DAPT (D5942) and Naloxone (N-004) were purchased from Sigma.

### Lentiviral transduction

Lentiviral particles containing BFP: pLVX-mTagBFP2-P2A-Puro (BIOSS Toolbox, University of Freiburg), GFP: pGIPZ (Openbiosystems, RHS4 346), and tdTomato: pLVX-tdTomato: (BIOSS Toolbox, University of Freiburg) as well as ERα construct (pHAGE-ESR1, Addgene, Plasmid #116737) were produced in HEK293 cells by mixing lentiviral vector and packaging vectors using branched polyethyleneimine (bPEI) (MW 25Kda, Sigma) as the transfection reagent. For transfection, 5 μg of DNA (4:3:1 of a transgene, pCMVdR8.74 (packaging plasmid; Addgene, Plasmid #22036) and pMD2.G (envelope plasmid, Addgene, Plasmid #12259) were diluted in 250 μL Opti-MEM (Invitrogen), afterward, 11.25 μL of bPEI (1 mg/mL) was added and incubated for 25 min at room temperature before transferring to HEK293 cells. 16 h after transfection, the medium was exchanged with the medium of the target cells. and 24 and 48 h after that, the media containing lentiviral particles were accumulated and filtered through a sterile 0.20 μm syringe filter (Millipore) to infect target cells. Infected cells include the human breast and pancreatic cancer cell lines, MSC, and HPMEC. Three days after transduction, infected cells were selected by adding puromycin (2 μg/mL for MDA-231, MCF-7, and Mia Paca-2; 4 μg/mL for PANC-1) (Sigma) to the culture medium in the case of BFP and by flow cytometry sorting in the case of GFP and tdTomato.

### Generation of STEMs and cancer spheroids

STEMs and cancer spheroids were generated by the hanging drop plate (Perfecta3D, HDP1096, USA). A 40 μl cell suspension was distributed into a hole of a 96-well hanging drop plate to form a hanging drop at a total density of 625 cells/µL by mixing cancer cell lines or MCF-10A; HPMEC, and MSC at a ratio of 5:3:2. The same cell density and ratio were applied to the spheroids comprising of breast cancer cells mixed with HPMEC or MSC. The choice of the cell ratio was based on our previous paper^28^. 4 ml of PBS was added to the peripheral reservoir, which prevented dehydration of the medium. The growth medium was prepared by mixing DMEM, endothelial cell medium, and alpha-MEM based on the ratio of 5:3:2 and exchanged every other day by taking 12 µl of mixed medium from a drop and adding 15 μl fresh mixed media to a drop. The plate was placed in an incubator at 37 °C and 5% CO_2_ for 10 days. The fluorescence images of STEMs and cancer spheroids formation were acquired by a Zeiss Cell Observer Z1 fluorescence microscope (Carl Zeiss, Germany). The extent of co-localization was quantified using Pearson’s correlation coefficient (PCC) acquired from ZEN blue 2.6 software (Carl Zeiss, Germany). In image analysis, PCC yields the correlation between pixels in two fluorescent images of the same field of view, thus, increasing overlapping fluorescent signals (photos) yields a value approaching 1, and those with no correlation yield negative values. Since PCC is based on pixel analysis, it can be applied to both high resolution and wide-field images. Confocal microscopy pictures were acquired using ZEISS LSM 710 Observer equipped with the software ZEN Black 2.3. For inhibition assay, STEMs were treated with DAPT solution or Naloxone solution on day 10 of STEMs incubation, and DMSO was added as a control, and the images were acquired 24 h later.

### Immunohistochemical staining

STEMs and spheroids were collected on day 10 and fixed in fresh 4% formaldehyde (Thermo Fisher Scientific) for 4 h at room temperature. STEMs and spheroids were then placed in 30% sucrose solution at 4 °C overnight or until they sink. Afterward, STEMs and spheroids were embedded in the O.C.T. compound (VWR, Germany) and cryosectioned by a cryo-stat (HYRAX C20, Zeiss). Cryosections (8 μm thick) on slides (Superfrost, VWR, Germany) were imaged using the fluorescence microscope. The extent of co-localization was quantified using Pearson’s correlation coefficient (PCC) acquired from ZEN blue 2.6 software. Sections were stained with hematoxylin & eosin (H&E) to visualize the histological morphology and structure. Activated Notch1 antibody (1:200, D3B8; #4147; Cell Signaling Technology) were used to stain the cryosections. In brief, cryosections were washed by PBS to remove the mounting media and then treated using heat-mediated antigen retrieval with pH 9 EDTA buffer (activated Notch-1) for 20 mins. Negative controls were completed by skipping the primary antibodies. Blocking was implemented for 1 h at room temperature with PBS containing 2.5% goat serum, 0.1% triton-X, 0.05% tween 20. Primary antibody incubation was carried out overnight at 4 °C. A goat anti-rabbit biotinylated secondary antibody (Vector Labs, 1:200) was used to detect the primary antibody and visualized using an HRP conjugated ABC system (VECTASTAIN Elite ABC kit; Vector Laboratories). DAB (Dako) was used as the chromogen to show brown color. The cryosections were then counterstained with Haematoxylin and mounted with Aquatex® (Merck). Bright-field images were acquired using a Zeiss Cell Observer-Z1 microscope.

### Immunofluorescence staining

For immunofluorescent staining, sections of STEMs were first blocked and then incubated with human Podocalyxin anti-rabbit (1:200, HPA002110; Sigma) overnight at 4 °C. Afterward, the sections were incubated by AlexaFluor-488 secondary antibody (Invitrogen, #A11029, 1:200) for 75 mins. The secondary antibody was then removed and the sections were washed twice with PBS before images were acquired.

### STEMs and spheroids transwell assay

Transwell migration assay was operated using 24-well cultured plates with 8 μm pore size membrane transwell inserts (Falcon). STEMs and spheroids were collected in a 150 μl serum-free mixed medium. Then they were placed on the upper compartment of chambers, while lower compartments were added with 500 μl of mixed medium containing 10% serum. Afterward, plates were incubated in the incubator until 30 h to allow cells to migrate. For transwell invasion assay, STEMs and spheroids were placed on the upper compartment of chambers that were coated with 100 μl of 500 μg/mL of Corning Matrigel matrix (Corning Cat. Nos. 354230) two hours earlier. Plates were then incubated until 30 h to allow cells to invade. Media were removed from the lower compartment and cells were fixed on the lower side of the inserts by 4% formaldehyde for 20 min incubation, and washed twice with PBS. The upper side of the membranes was carefully swabbed and inserts were air-dried. Cells expressing fluorescence protein on the lower side of the membrane were imaged and counted using ImageJ.

### Cells transwell assay

MSC and HPMEC were trypsinized and resuspended in a serum-free medium. 15,000 cells were placed on an insert of a transwell chamber for cell migration. The same amounts of cells were added onto an insert that was coated with 100 μl of 500 μg/mL of Corning Matrigel matrix for cell invasion, while the lower compartments were added with 500 μL of media containing 10% FBS. Plates were then incubated for 30 h to allow cells to migrate or invade. Afterward, the transwell membranes were fixed with 4% formaldehyde for 20 min. Five fields per membrane were imaged and cells were counted using ImageJ.

### Effect of mixed medium or fluorescent proteins on the cell phenotypes

Transfected and non-transfected MDA, MSC, and HPMEC were treated by mix medium or their respective culture media for 24 h prior to the trypsinization. On second day, all the cells were trypsinized and resuspended in a serum-free mix medium or their respective culture media. 15,000 cells were placed on an insert of a transwell chamber for cell migration, while the lower compartments were added with 500 μL of mix media or their respective culture media containing 10% FBS. Plates were then incubated for 30 h to allow cells to migrate. Afterward, the transwell membranes were fixed with 4% formaldehyde for 20 min. DAPI (Cat No. H-1500) was used to identify migrated cells. Five fields per membrane were imaged and cells were counted using ImageJ.

### 3D invasion assays

After 10 days of culture, MDA and MCF STEMs were harvested and implanted in the center of each well of a 96-well plate, and coated with a 2 mg/ml Matrigel mixture (one STEMs per well in 100 μl of Matrigel per well). The Matrigel mixture was prepared by mixing 20 μl of Corning Matrigel matrix (10 mg/ml) with 80 μl of mix medium without FBS. After STEMs embedding, the plate was incubated for 2 hours in the incubator to solidify the gels. Thereafter 100 μl of mixed medium with 10% FBS was overlaid on the Matrigel matrix in each well. The invasion system was incubated for 2 days and images were taken on day 0 and day 2 by fluorescence microscope after embedding.

### Collective migration of MDA STEMs in a 3D-Bioprinted model system

To investigate if MDA STEMs could recapitulate metastatic events associated with the primary tumor, we printed a construct comprising of two conjoined concentric rings and a center disk laden with the fetal bovine serum to establish a radial gradient of chemokine. The inner concentric ring was created using type I collagen (Advanced Biomatrix, Catalog: 5153), and the MDA STEMs (10–15 per ring) were printed in the outer concentric ring using carboxylated agarose (CA) hydrogel-based bioink (CA bioink). CA was synthesized based on our previous work^78^. The CA bioink formulation was prepared as follows. Lyophilized 60% Carboxylated agarose (CA60) (261.25 mg) and native agarose (NA) (Thermo Fischer) (13.75 mg) were added into 2.3 mL PBS and the mixtures was heated up to 95 °C until a clear solution was obtained, and then transferred into a 42 °C oven for at least 10 min. Afterward, MDA STEMs were collected and mixed with CA bioink equilibrated at 42 °C. The MDA STEMs-laden CA bioink was then transferred to a 37 °C cartridge for printing outer concentric rings on an Inkredible-2 (Cellink, Sweden) 3D printer. The same CA bioink formulation was mixed with FBS to produce the 10% FBS-laden CA bioink, which was used to print center disks. The g-code was produced on Slic3r (GNU Affero General Public License) and then edited on Cellink HeartWare (Cellink, Sweden). After an outer concentric ring and a center disk were assembled in a well of a 24 well plate, type I Collagen prepared according to manufacturer’s instructions was introduced between the outer ring and disk. Afterward, the plates were incubated at 37 °C for 1 h and inner concentric rings were formed between the outer ring and disk. Mix medium without FBS was given to the printed structures that were then cultured in the incubator. Bright-field and fluorescent images were acquired using a Zeiss Cell Observer-Z1 microscope.

### Western blotting

The cells were lysed in Laemmli buffer (RIPA) with 1% phosphatase protease inhibitor (Thermo Fisher Scientific), and protein concentration for each sample was determined by a Pierce BCA protein assay kit (Thermo Fisher Scientific). Samples containing 20 μg of protein were cooked and loaded in an 8% (wt/vol) polyacrylamide/SDS gel, and then transferred onto nitrocellulose membranes (Bio-Rad). Afterward, the membranes were immediately blocked with 5% (w/v) BSA diluted in TBST for 1 h at room temperature. ERα antibody (Santa Cruz Biotechnology, D-12,1:500) was diluted in 2.5% BSA in TBST and then added to probe. GAPDH (Santa Cruz Biotechnology, sc-25778, 1:500) was used as a loading control. Blots were developed using HRP-conjugated secondary antibodies (Thermo Fisher Scientific, #31430, 1:2500) against the species of the primary antibody and imaged by SuperSignal West Pico PLUS chemiluminescence system (Thermo Fisher Scientific).

### Chorioallantoic membrane (CAM) assay

Fertilized chicken eggs were obtained from LSL Rhein-Main in Germany and were carefully wiped with rough paper towels to remove dirt, feathers, and excrement and then incubated for 2 days in an incubator set at 37 °C and 65% humidity. Eggs were placed horizontally in an incubator with a moving tray and rotated three times a day continuously to avoid the yolk and embryo sticking to the shell. On day 3, eggs were cracked, the yolk with embryo and egg white were kept close to the bottom of square plastic weighing boats which had been sterilized. *Ex ovo* cultures were reincubated under the same conditions until day 7 when plastic rings obtained from inoculation loops (Sarstedt) were carefully placed at blood vessel crossings forming a Y shape to delimitate the samples at places providing enough nutrition. In parallel, STEMs were collected and dispersed in a 30 µl of 50% mix medium + 50% Matrigel and then added to the ring. Embryo and tumor growth were allowed further for 6 more days until day 13 before the samples were excised. An upright microscope (Mustool, G1200, China) was used to capture the images on day 1, 3, and 6. In the European Union countries, CAM assay is not considered an animal experiment and, therefore, does not require ethical approval.

### Histopathological analysis of CAM tumors

Samples were excised away from the embryos before being were fixed in 10% formalin for 24 h, washed with 70% ethanol, and embedded in paraffin. 4 μm sections were cut and stained for HE. Immunohistochemical analysis was performed by using reagents specific for CK18 (Clone B23.1, prediluted, Ventana), CK22 (Clone MM-1012-02, prediluted, Immuno Bio), Calponin (Clone 760-4376, 1:2000, Ventana), CD34 (Clone QBEnd/10, prediluted, Ventana), Ki-67 (Clone IR626, prediluted, Dako), ERα (Clone SP1, prediluted, Ventana), PR (Clone 1E2, prediluted, Ventana), Her2 (Clone 4B5, prediluted, Ventana), p63 (Clone 4A4, prediluted, Ventana), human mitochondria (MAB1273, 1:200, Millipore), CD106 (MA5-16429, 1:100, Thermo Fisher) on the BenchMark ULTRA IHC/ISH System. For all immunohistochemical stainings, DAB was used as a chromogen.

### Statistics and reproducibility

The quantitative data presented in this study were acquired from at least three independent cultures. Statistical analyses were performed by one-way ANOVA statistical package and statistical significance was calculated using Tukey’s multiple comparison tests. Statistical significance was set as ***P < 0.001; **P < 0.01; *P < 0.05. All calculations were performed using OriginPro 2022 (OriginLab Corporation, MA, USA). Sample sizes and replicates are described in legends of Figures.

### Reporting summary

Further information on research design is available in the Nature Research Reporting Summary linked to this article.

## Supplementary information

Supplementary Information-New

Description of Additional Supplementary Data

Supplementary data

Supplementary Movie 1

Supplementary Movie 2

Supplementary Movie 3

Supplementary Movie 4

Supplementary Movie 5

Supplementary Movie 6

Supplementary Movie 7

Reporting summary

## Data Availability

All data necessary to support our conclusions are included in the main manuscript and the supplementary information files. All source data are available within the Supplementary Data. Unprocessed gels of Fig. 4a and Supplementary Fig. 13a shows in Supplementary Figs. 21 and 22. Information of Packaging plasmid #22036, envelope plasmid #12259 can be accessed at Addgene website. All other data are available from the corresponding author on reasonable request.
