# Supplementary material for: Mesenchymal-endothelial nexus in breast cancer spheroids induces vasculogenesis and local invasion in a CAM model
Source: Commun Biol. 2022 Nov 27;5:1303. doi: 10.1038/s42003-022-04236-5 (PMC9701219; doi:10.1038/s42003-022-04236-5)
Supplement: Supplementary file 1 — Supplementary Information-New [file 42003_2022_4236_MOESM1_ESM.pdf]

Supplementary Information for

Mesenchymal-Endothelial Nexus in Breast Cancer Spheroids Induces Vasculogenesis and Local Invasion in a CAM Model

Aijun Li[1], Simone Muenst[2], Julius Hoffman[1], Laurent Starck[1], Melika Sarem[1], Andreas Fischer[3,4], Gregor Hutter[5,6], V. Prasad Shastri[1,7,*]

[1]Institute for Macromolecular Chemistry, University of Freiburg, Freiburg, 79104, Germany

[2]Institute for Medical Genetics and Pathology, University Hospital Basel, Basel 4031, Switzerland

[3]Institute for Clinical Chemistry, Göttingen University Hospital, 37075 Göttingen, Germany

[4]Division Vascular Signaling and Cancer, German Cancer Research Center, 69120 Heidelberg, Germany

[5]Brain Tumor Immunotherapy Lab, Department of Biomedicine, University of Basel, Basel 4031, Switzerland

[6]Department of Neurosurgery, University Hospital Basel, Basel, Basel 4031, Switzerland

[7]BIOSS - Centre for Biological Signalling Studies, University of Freiburg, Freiburg, 79104, Germany

*Corresponding author: V. Prasad Shastri

Email: prasad.shastri@gmail.com, prasad.shastri@makro.uni-freiburg.de

Supplementary Figure 1 to 22 and Table 1

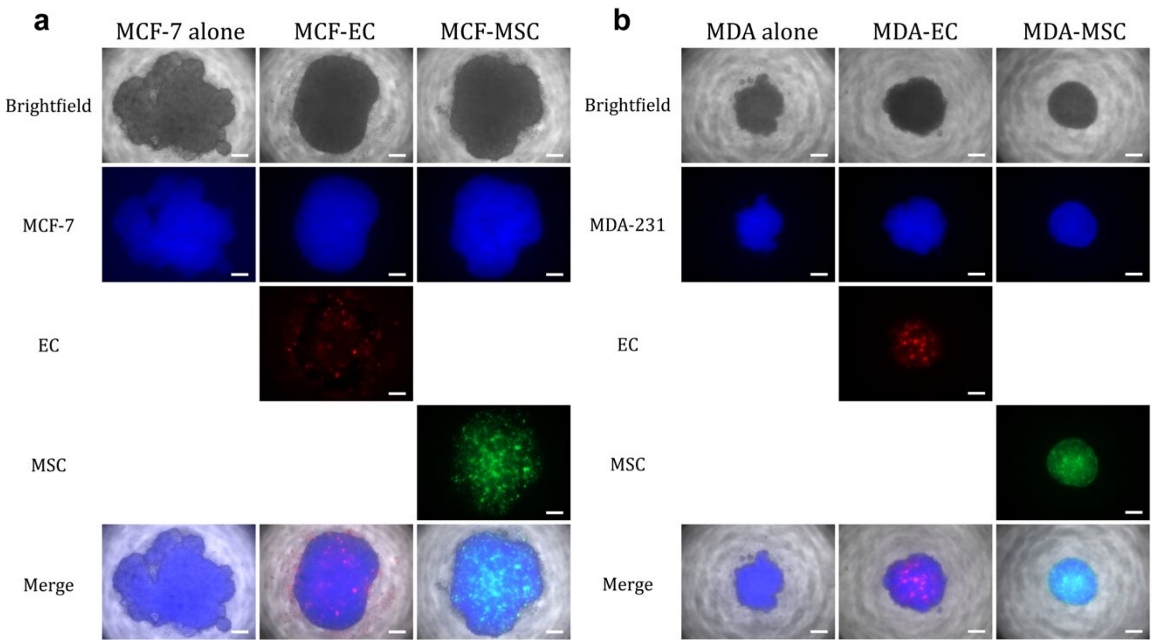

Supplementary Figure 1: Absence of MSC or EC alters cellular organization in the STEMs: **(a)** Brightfield and fluorescence microscopy images (MCF-7/BFP, EC/tdTomato, and MSC/GFP) of spheroids of MCF alone, MCF-EC and MCF-MSC at day 10, scale bar: 200 µm. **(b)** Brightfield and fluorescence microscopy images (MDA-231/BFP, EC/tdTomato, and MSC/GFP) of spheroids of MDA alone, MDA-EC, and MDA-MSC, scale bar: 200 µm.

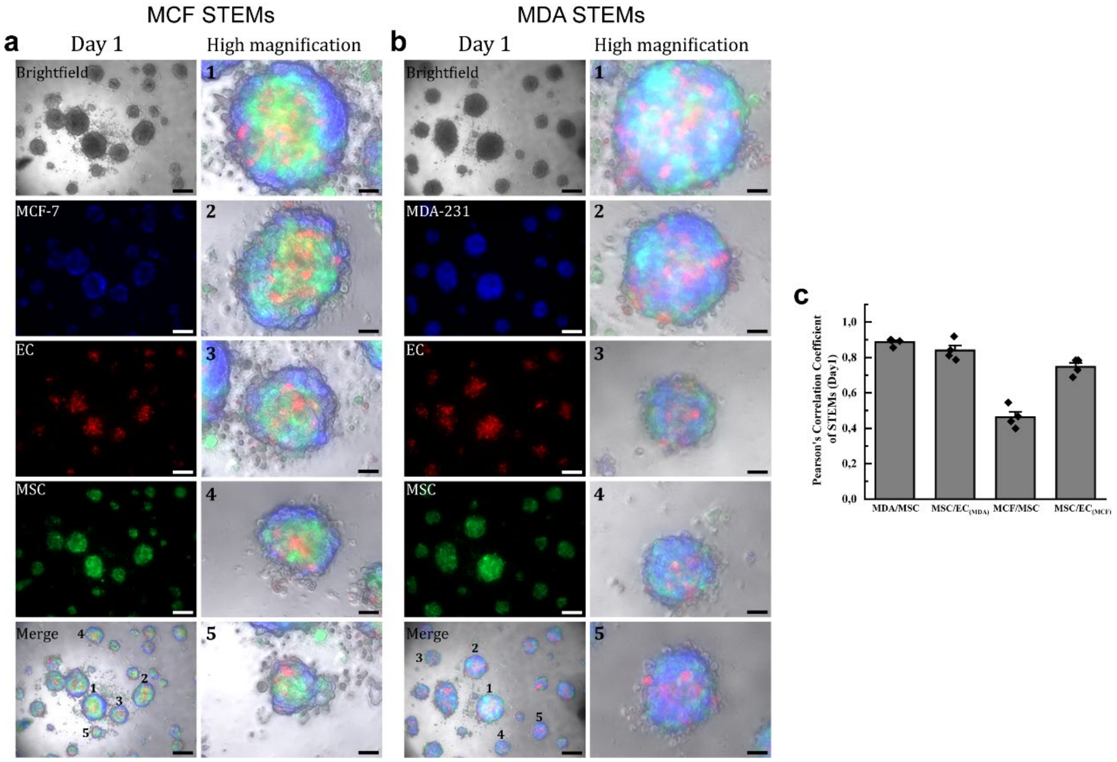

Supplementary Figure 2: MSC-EC interactions define the organization of BCC in STEMs within 24 hours: (a) Brightfield and fluorescence microscopy images (MCF-7/BFP, EC/tdTomato, and MSC/GFP) of MCF STEMs, scale bar: 200 µm. Corresponding high magnification merge images are shown in the right column (Scale bar: 50 µm). **(b)** Brightfield and fluorescence microscopy images (MDA-231/BFP, EC/tdTomato, and MSC/GFP) of MDA STEMs, scale bar: 200 µm. Corresponding high magnification merge images are shown in the right column (Scale bar: 50 µm). **(c)** PCCs of MDA/MSC, MSC/$EC_{(MDA)}$, MCF/MSC, MSC/$EC_{(MCF)}$ of STEMs (Day 1), n = 4.

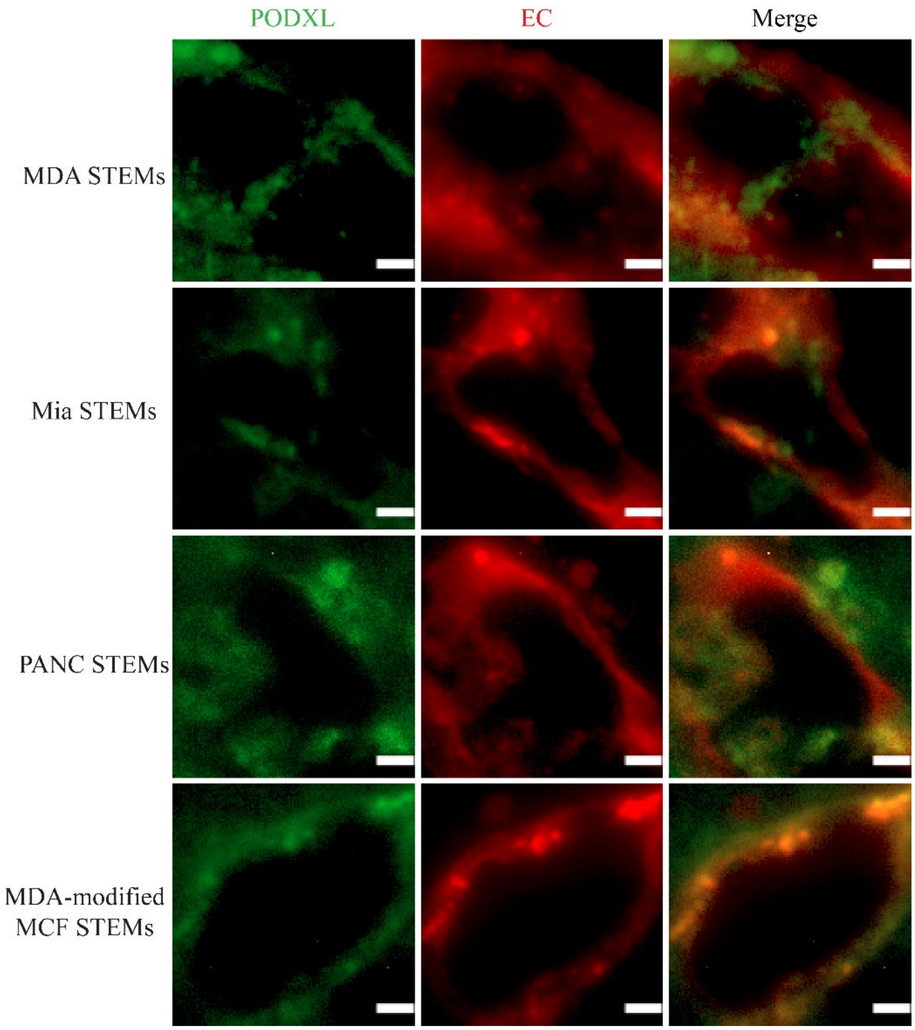

Supplementary Figure 3: PODXL marker for vasculature in various STEMs. EC/tdTomato structures with lumen morphology stained for PODXL (green) in MDA, Mia, PANC, and 40% MDA-modified MCF STEMs, scale bar: 5 µm.

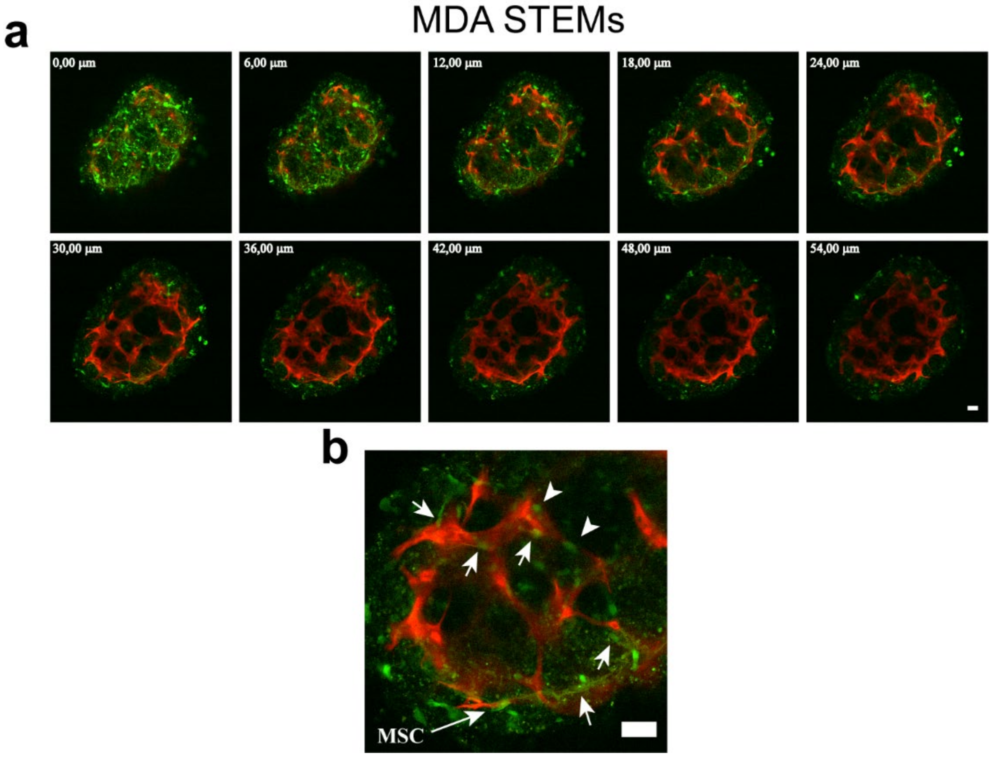

Supplementary Figure 4: Relative position between EC vasculature and MSC in MDA STEMs: **(a)** Confocal Z-stacks (thickness: 54 µm, 6 µm/slice) showing relative position of EC (tdTomato fluorescence) and MSC (GFP fluorescence) in the MDA STEMs. **(b)** An exemplar image from confocal Z-stacks with high magnification, indicating the close relationship between MSC/GFP and EC/tdTomato vasculature in the MDA STEMs, white arrows indicate MSC in close association to the EC structures. Scale bar: 50 µm.

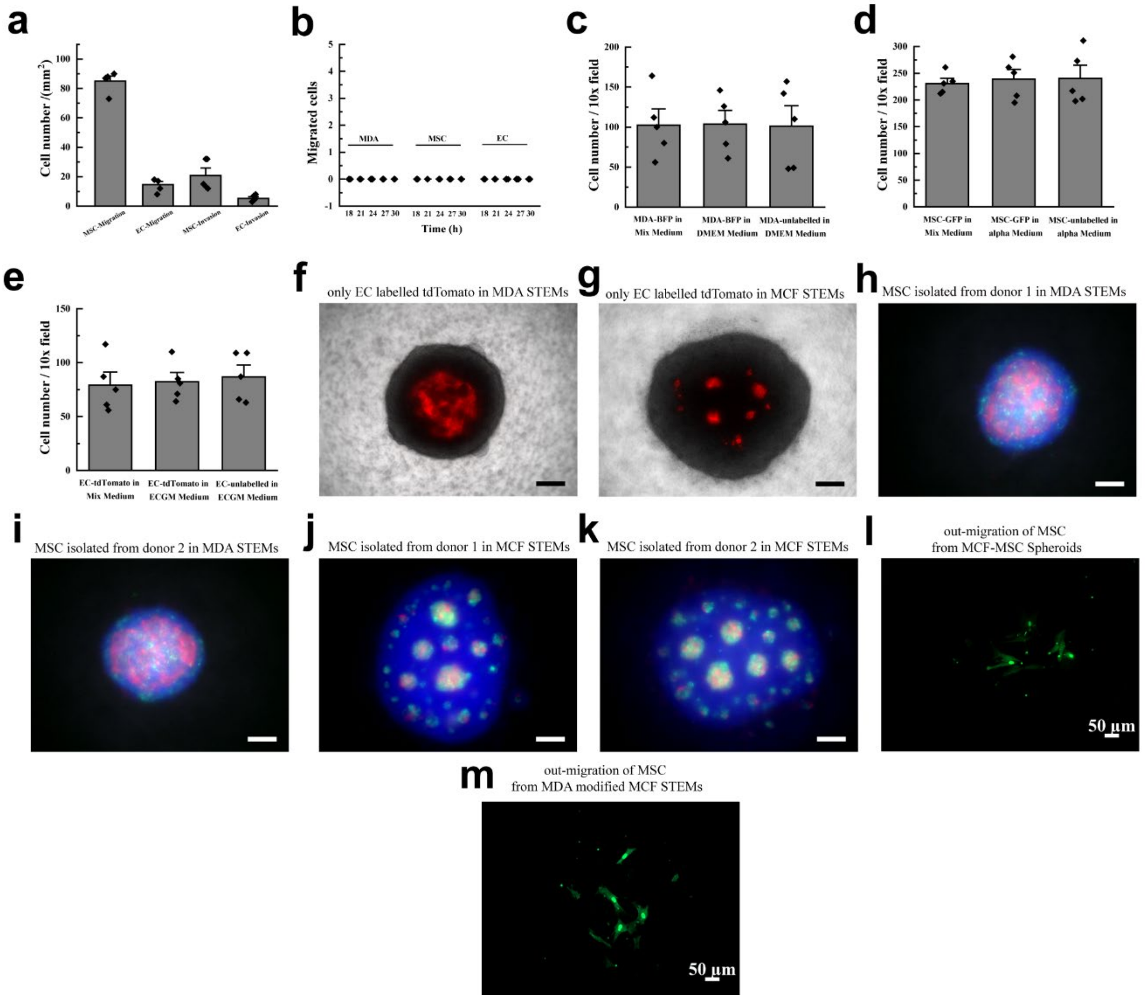

Supplementary Figure 5: **(a)** Quantification of migration and invasion of MSC and EC after 30h in transwell chambers, confirming the migratory potential of MSC and EC in 2D culture. n = 5 **(b)** Quantification of cell migration over time from MCF STEMs in transwell chambers, n = 4. No out-migration of any cell population from MCF STEMs is observed. **(c)** Quantification of migration of MDA-BFP (transfected) in mix medium or DMEM, and MDA (un-transfected) in DMEM, after 30h in transwell chambers, n = 5, P value between MDA-BFP in Mix Medium and MDA-BFP in DMEM Medium is 0.96093, P value between MDA-unlabelled in DMEM Medium and MDA-BFP in DMEM Medium is 0.93262. **(d)** Quantification of migration of MSC-GFP (transfected) in mix medium or alpha-MEM, and MSC (un-transfected) in alpha MEM, after 30h in transwell chambers, n = 5, P value between MSC-GFP in Mix Medium and MSC-GFP in alpha Medium is 0.66132, P value between MSC-unlabelled in alpha Medium and MSC-GFP in alpha Medium is 0.97191. **(e)** Quantification of migration of EC-tdTomato (transfected) in mix medium or ECGM, and EC (un-transfected) in ECGM, after 30h in transwell chambers, n = 5, P value between EC-tdTomato in Mix Medium and EC-tdTomato in ECGM Medium is 0.82903, P value between EC-unlabelled in ECGM Medium and EC-tdTomato in ECGM Medium is 0.72649. **(f)** **(g)** Merged brightfield and fluorescence microscopy images of EC/tdTomato in MDA STEMs (MDA/un-transfected and MSC/un-transfected) and MCF STEMs (MCF/un-transfected and MSC/un-transfected), scale bar: 200 µm. EC show the same organization as EC in the MDA and MCF STEMs comprised of all cells expressing fluorescent proteins. Fluorescence microscopy images of MDA STEMs **(h)** **(i)** and MCF

STEMs **(j)** **(k)** prepared using MSC isolated from two additional different donors showing the lack of donor dependency, scale bar: 200 µm. **(l)** Fluorescence microscopy image of MSC migration across the transwell membrane from MCF-MSC spheroid after 21 h incubation. MSC showed a migratory phenotype after removal of EC from MCF-STEMs. **(m)** Fluorescence microscopy image of MSC migration in the transwell membrane from MDA-modified MCF STEMs (40%MDA+10%MCF) after 21 h incubation. Data in bar plots show mean values ± SD.

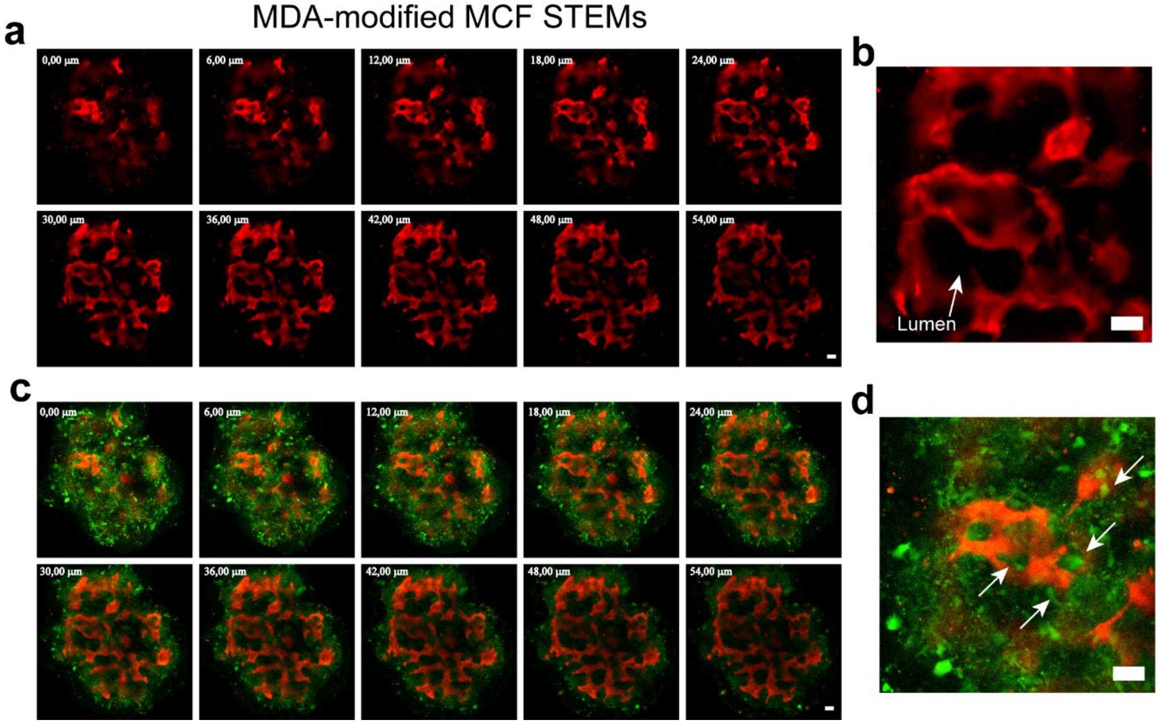

Supplementary Figure 6: MDA promotes reorganization of EC into vascular structures and the relative position between EC structure and MSC in MDA-modified MCF-STEMs: (a) Confocal Z-stacks (thickness: 54 µm, 6 µm/slice) showing organization of EC (tdTomato fluorescence) in the 40% MDA-modified MCF STEMs. **(b)** An exemplar image from confocal Z-stacks with high magnification in the MDA-modified MCF STEMs, EC/tdTomato organization shows clear lumen structure (white arrows). **(c)** Confocal Z-stacks (thickness: 54 µm, 6 µm/slice) showing relative position of EC (tdTomato fluorescence) and MSC (GFP fluorescence) in the MDA-modified MCF STEMs. **(d)** An exemplar image from confocal Z-stacks with high magnification, indicating the close relationship between MSC/GFP and EC/tdTomato vasculature in the MDA-modified MCF STEMs, white arrows indicate MSC in close association to the EC structures. Scale bar: 50 µm.

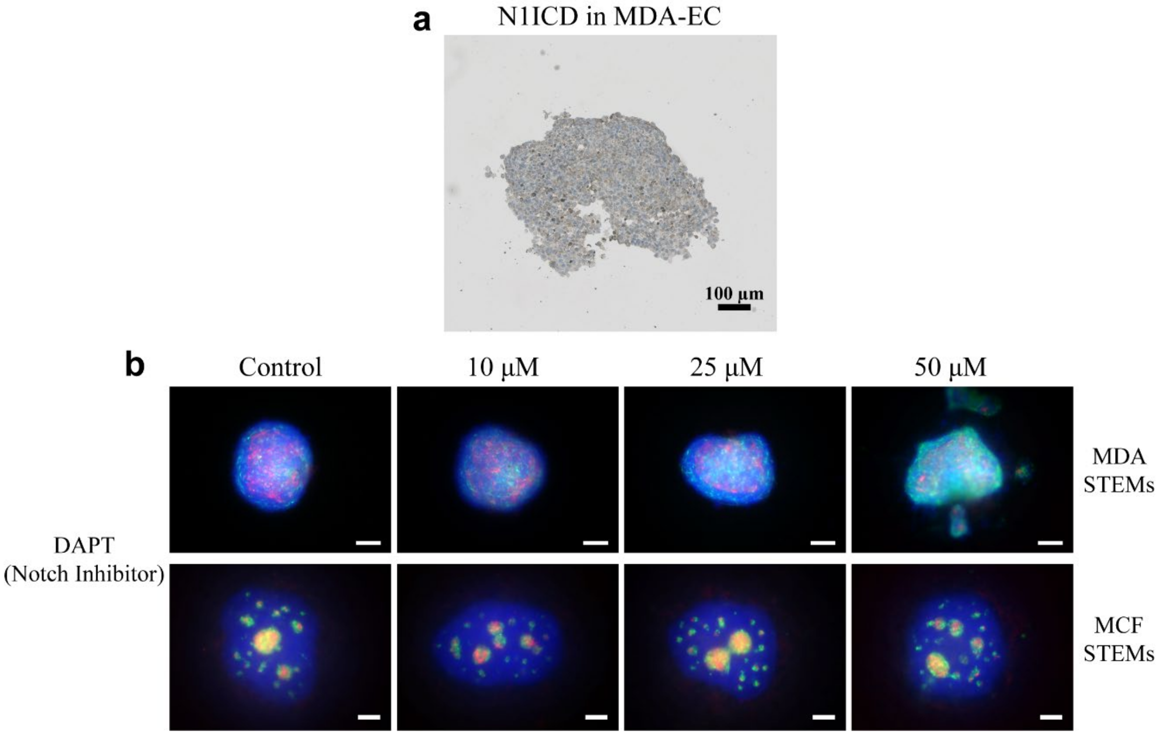

Supplementary Figure 7: Immunostaining for N1ICD in MDA-EC spheroid and effect of DAPT on MDA and MCF STEMs. (a) Immunohistochemical staining for N1ICD (activated NOTCH) in MDA-EC spheroid confirming that the absence of MSC abrogates activation NOTCH1. **(b)** Composite multichannel fluorescence images of MDA and MCF STEMs after the treatment of DAPT (10 µM to 50 µM) for 24 hours. Lower concentrations (10 µM, 25 µM) of DAPT have no discernible effect on MDA and MCF STEMs, exposure to 50 µM of DAPT led to fragmentation and blebbing in MDA STEMs and not in MCF STEMs, scale bar: 200 µm.

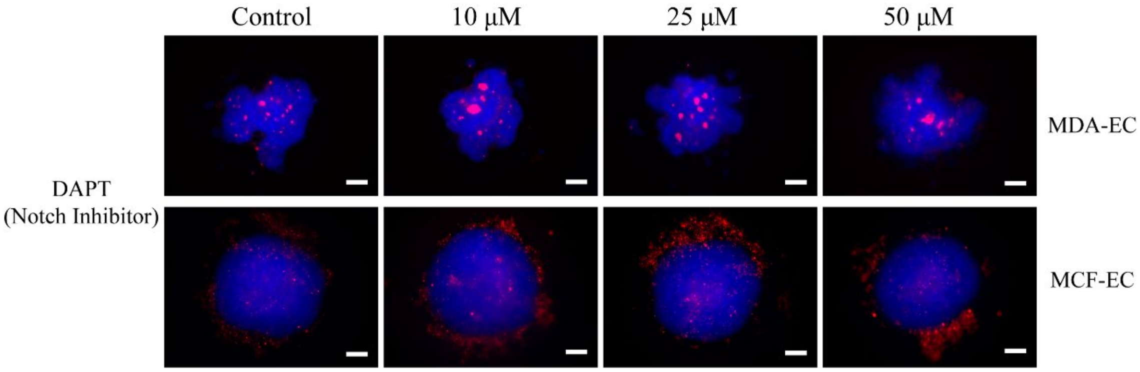

Supplementary Figure 8: Lack of the effect of DAPT on MDA-EC and MCF-EC spheroids. Composite multichannel fluorescence images of MDA-EC and MCF-EC after the treatment of DAPT (10 μM to 50 μM) for 24 hours. DAPT has been shown no discernible effect on MDA-EC and MCF-EC. Scale bar: 200 μm.

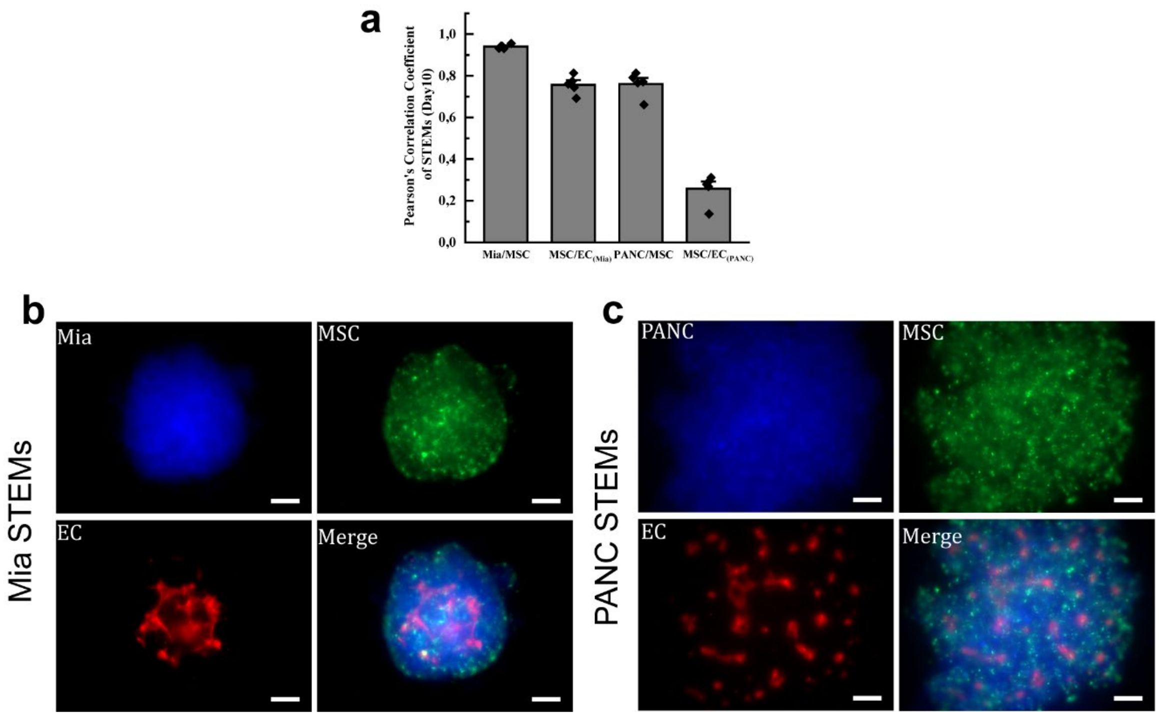

Supplementary Figure 9: EC organization is formed in the ERα-negative pancreatic cancer cell lines Mia-PaCa-2 and PANC-1 STEMs. (a) PCCs of Mia/MSC, MSC/EC$_{(Mia)}$, PANC/MSC, MSC/EC$_{(PANC)}$ in STEMs, n = 5. **(b and c)** Fluorescence microscopy images of Mia STEMs (Mia Paca-2/BFP, EC/tdTomato, MSC/GFP) and PANC STEMs (PANC-1/BFP, EC/tdTomato, MSC/GFP), showing the organization of EC into structures and the uniform distribution of MSC similar to what is observed in MDA STEMs. Scale bar: 200 µm.

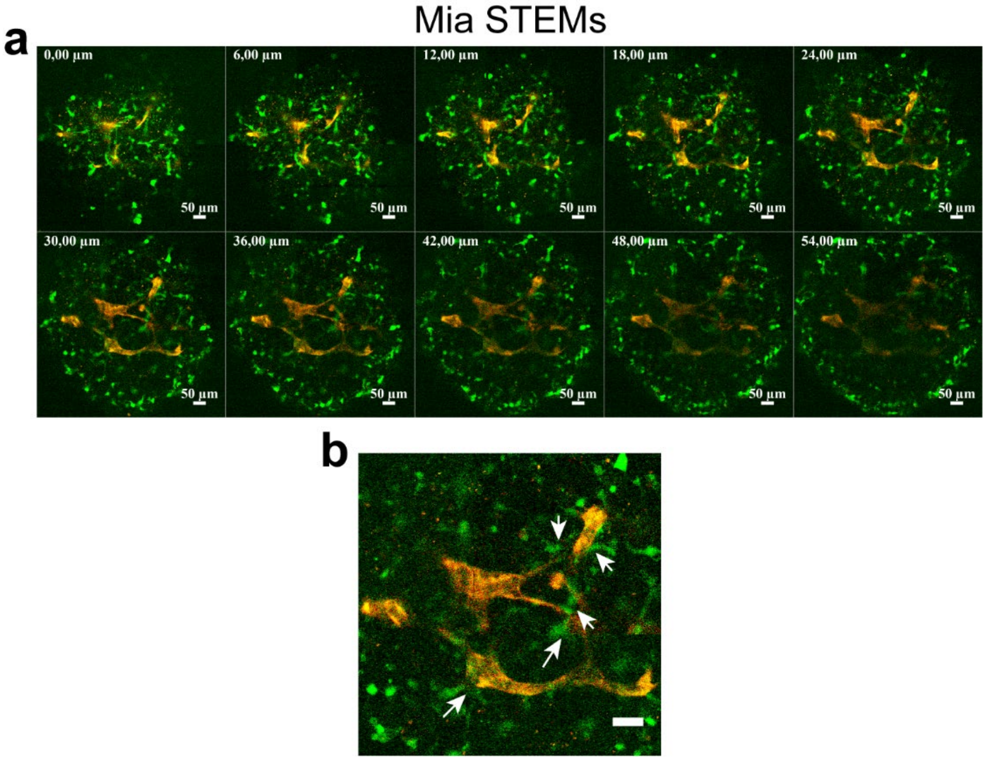

Supplementary Figure 10: Relative position between EC vasculature and MSC in Mia STEMs: **(a)** Confocal Z-stacks (thickness: 54 μm, 6 μm/slice) showing relative position of EC (tdTomato fluorescence) and MSC (GFP fluorescence) in the Mia STEMs. **(b)** An exemplar image from confocal Z-stacks with high magnification, indicating the close relationship between MSC/GFP and EC/tdTomato vasculature in the Mia STEMs, white arrows indicate MSC in close association to the EC structures. Scale bar: 50 μm.

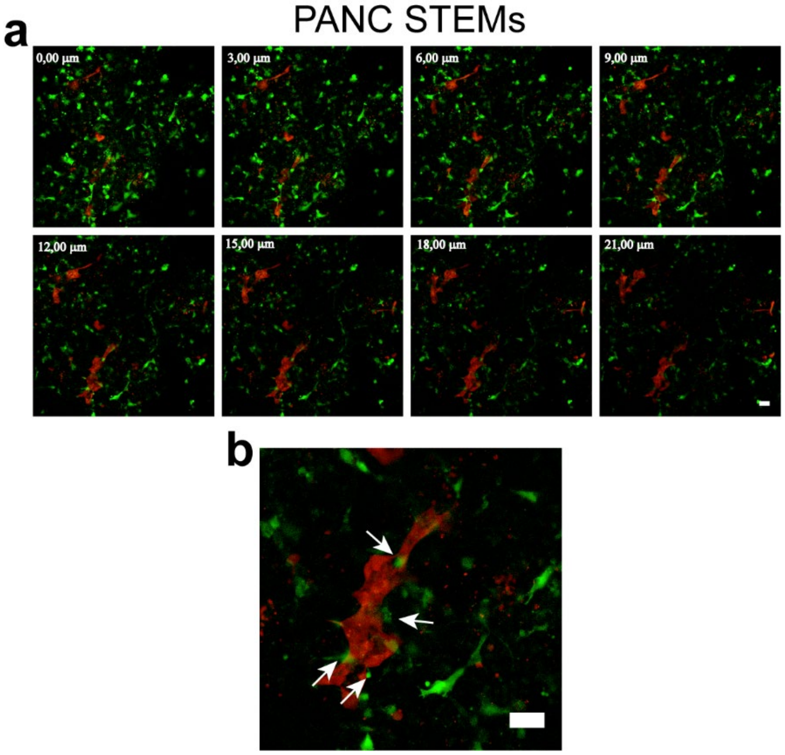

Supplementary Figure 11: Relative position between EC vasculature and MSC in PANC STEMs: (a) Confocal Z-stacks (thickness: 21 µm, 3 µm/slice) showing relative position of EC (tdTomato fluorescence) and MSC (GFP fluorescence) in the PANC STEMs. **(b)** An exemplar image from confocal Z-stacks with high magnification, indicating the close relationship between MSC/GFP and EC/tdTomato vasculature in the PANC STEMs, white arrows indicate MSC in close association to the EC structures. Scale bar: 50 µm.

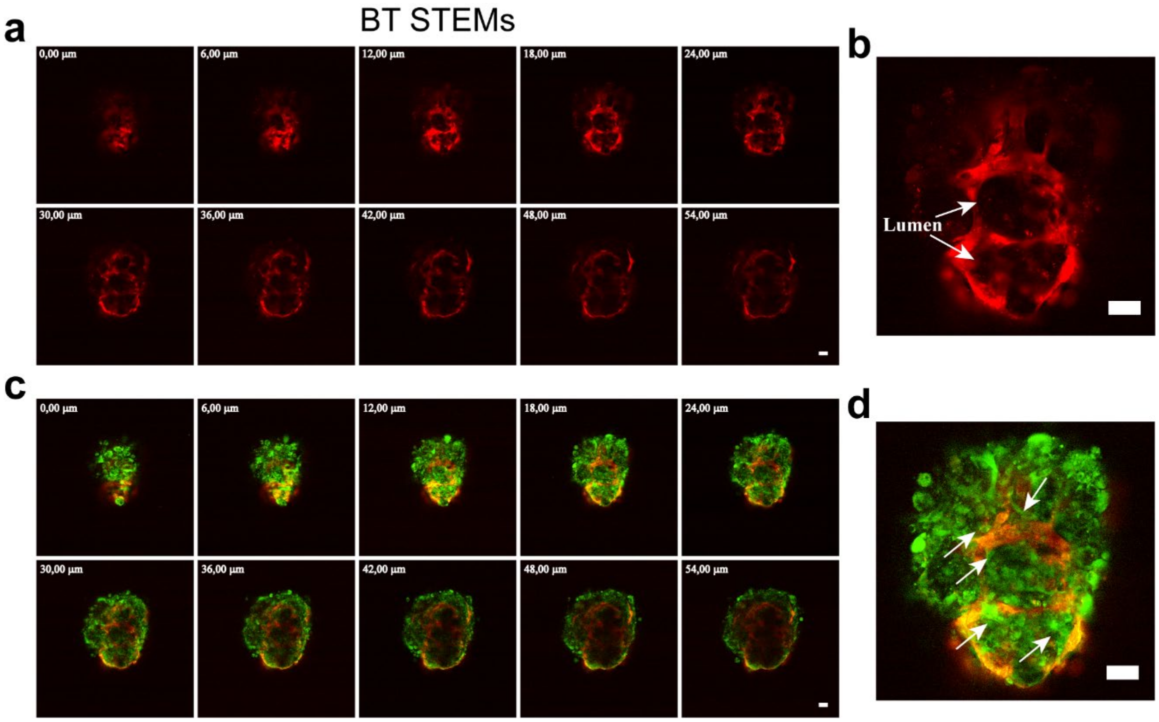

Supplementary Figure 12: EC organization in BT SETMs and relative position between EC vasculature and MSC in BT STEMs: (a) Confocal Z-stacks (thickness: 54 µm, 6 µm/slice) showing organization of EC (tdTomato fluorescence) in the BT STEMs (BT549/EC/MSC). **(b)** An exemplar image from confocal Z-stacks with high magnification in the BT STEMs, EC/tdTomato organization shows clear lumen structure (white arrows). **(c)** Confocal Z-stacks (thickness: 54 µm, 6 µm/slice) showing relative position of EC (tdTomato fluorescence) and MSC (GFP fluorescence) in the BT STEMs. **(d)** An exemplar image from confocal Z-stacks with high magnification, indicating the close relationship between MSC/GFP and EC/tdTomato vasculature in BT STEMs, white arrows indicate MSC in close association to the EC structures. Scale bar: 50 µm.

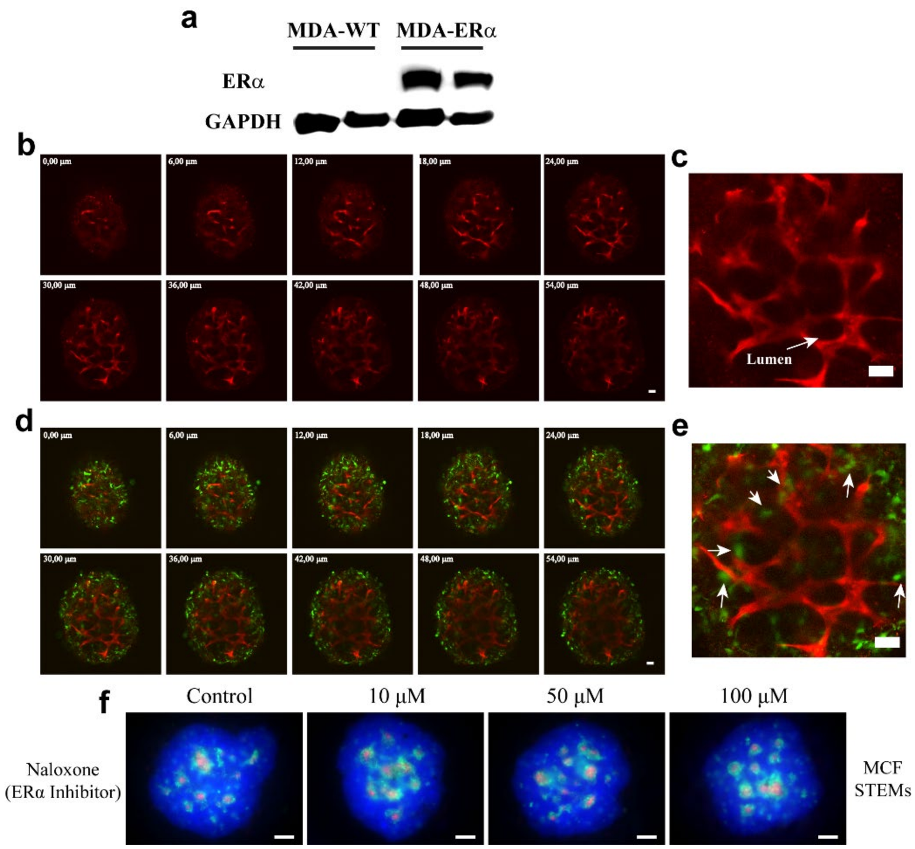

Supplementary Figure 13: Knock-in of ERα has no discernible effect on the EC organization of MDA STEMs and relative position between EC vasculature and MSC in MDA-ERα STEMs: **(a)** Immunoblot analysis of ERα from total cell protein lysates of MDA-MB-231 transduced with scrambled DNA sequence (WT) or a DNA sequence targeting ERα, using antibody D-12 revealed a 66 kDa band confirming the expression status of ERα in MDA. GAPDH was used as a loading control. **(b)** Confocal Z-stacks (thickness: 54 µm, 6 µm/slice) showing organization of EC (tdTomato fluorescence) in the MDA-ERα STEMs. **(c)** An exemplar image from confocal Z-stacks with high magnification in the MDA-ERα STEMs, EC/tdTomato organization shows clear lumen structure (white arrows). **(d)** Confocal Z-stacks (thickness: 54 µm, 6 µm/slice) showing relative position of EC (tdTomato fluorescence) and MSC (GFP fluorescence) in the MDA-ERα STEMs. **(e)** An exemplar image from confocal Z-stacks with high magnification, indicating the close relationship between MSC/GFP and EC/tdTomato vasculature in the MDA-ERα STEMs, white arrows indicate MSC in close association to the EC structures. Scale bar: 50 µm. **(f)** Effect of Naloxone on the cellular organization in MCF STEMs. MCF STEMs were treated with Naloxone from 10 µM to 100 µM for 24 hours. Naloxone did not impact EC organization and cellular organization in general, this suggests that loss of ERα expression in BCC might be part of a larger phenotype transformation paradigm and an important step in tumor-angiogenesis. Scale bar: 200 µm.

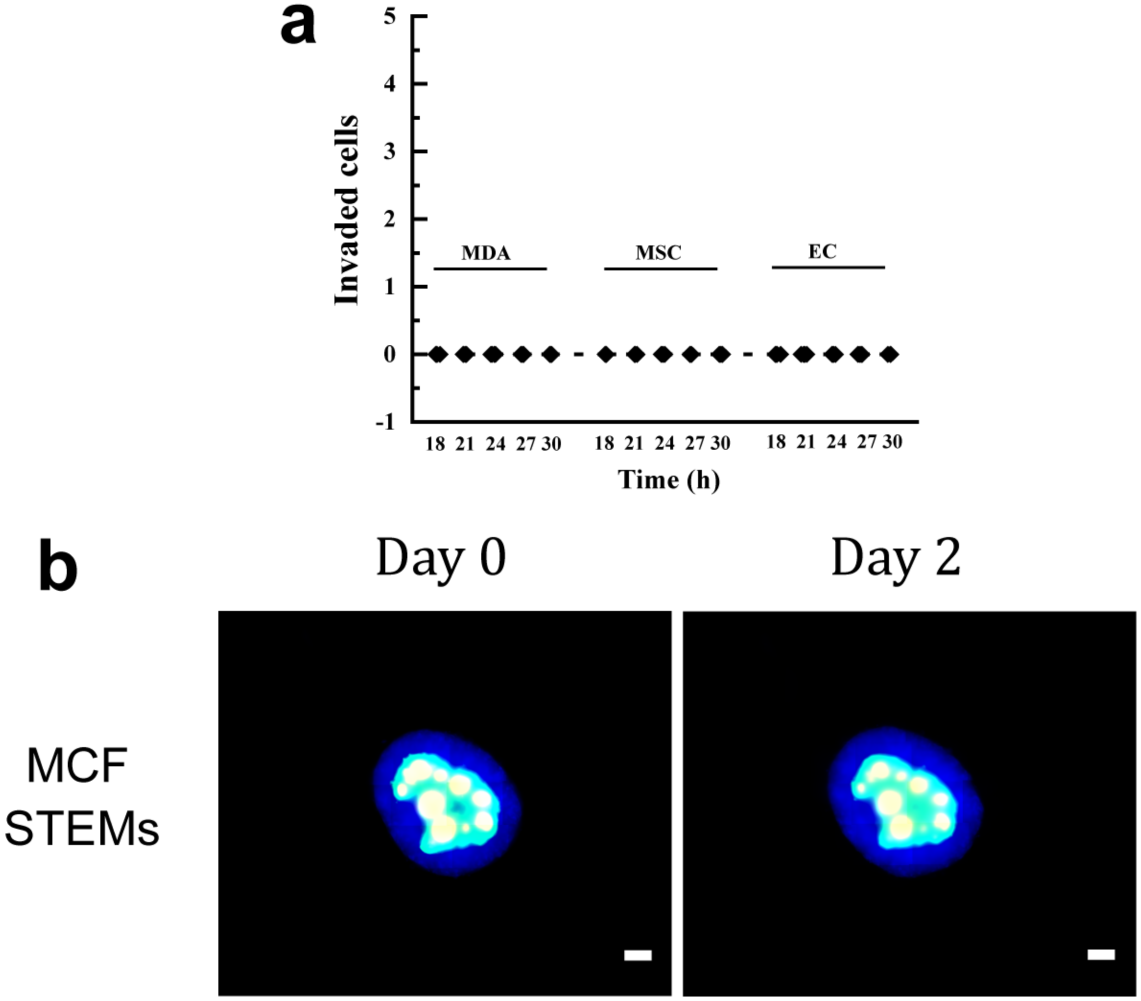

Supplementary Figure 14: No invasive behavior of MCF STEMs: (a) Quantification of cell invasion over time from MCF STEMs into Matrigel in transwell chambers, n = 4. **(b)** Fluorescence microscopy images of MCF STEMs invading into Matrigel, scale bar: 200 µm. Both A and B show no invasion of any cell population from MCF STEMs.

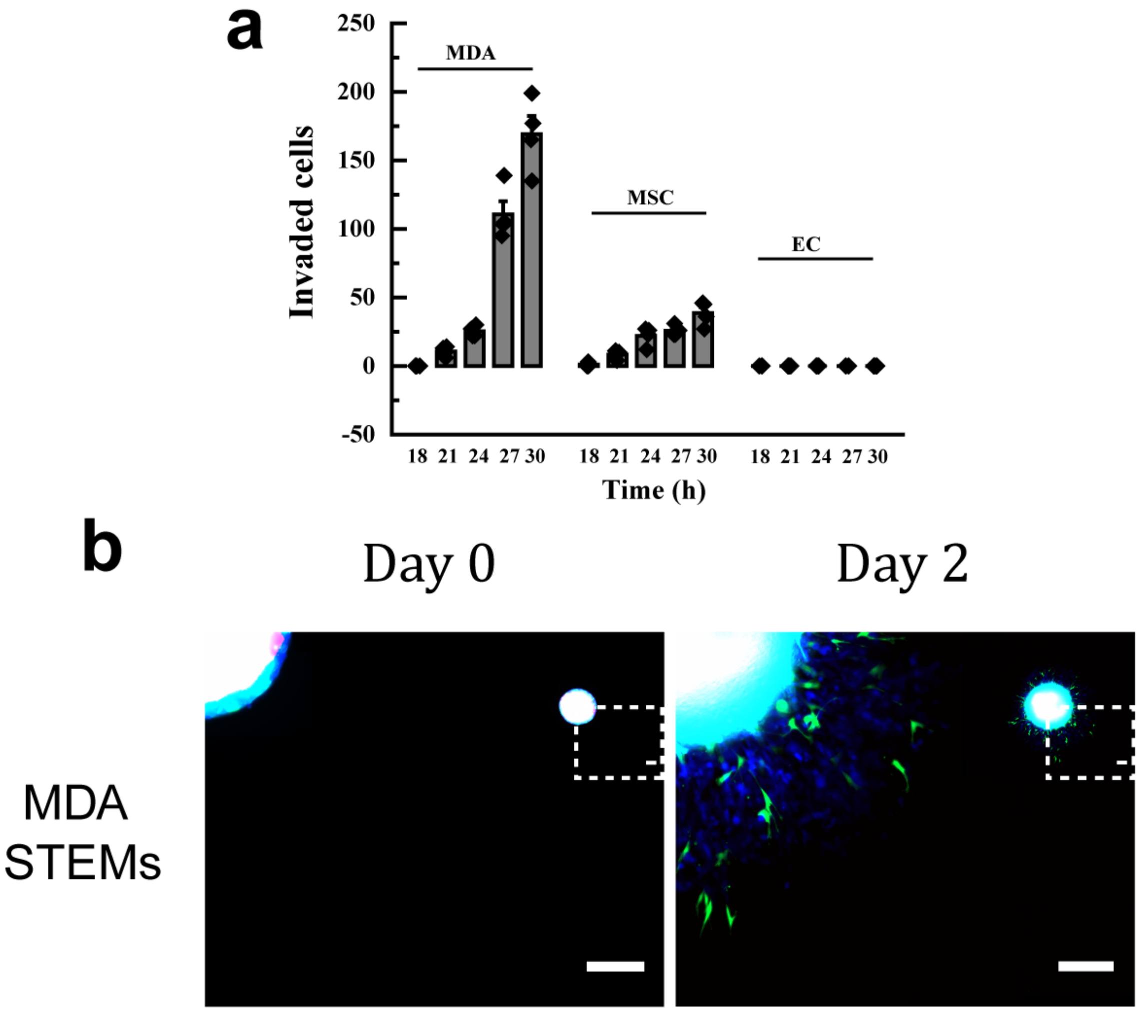

Supplementary Figure 15: MDA STEMs show invasive behavior: (a) Quantification of cell invasion over time from MDA STEMs into Matrigel in transwell chambers, n = 4. **(b)** Fluorescence microscopy images of MDA STEMs invading into Matrigel showing a leading-edge dominated by MSC. The main images are high magnification of the dashed box area, scale bar: 200 μm.

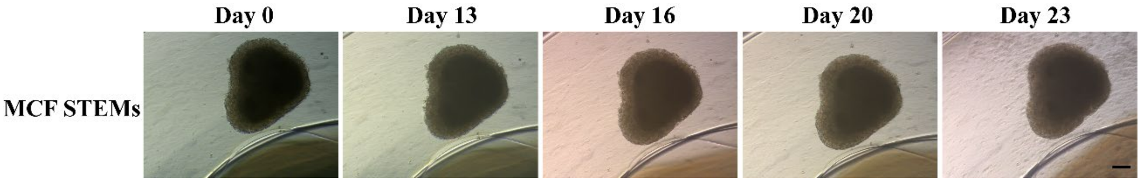

Supplementary Figure 16: MCF STEMs do not exhibit collective migration in a 3D-Bioprinted model system. Image sequences shows that no cell cluster (tumor metastatic unit) from MCF STEMs was formed, which is consistent with the inherent non-invasive capacity of MCF STEMs as previous results showed, scale bar: 200μm.

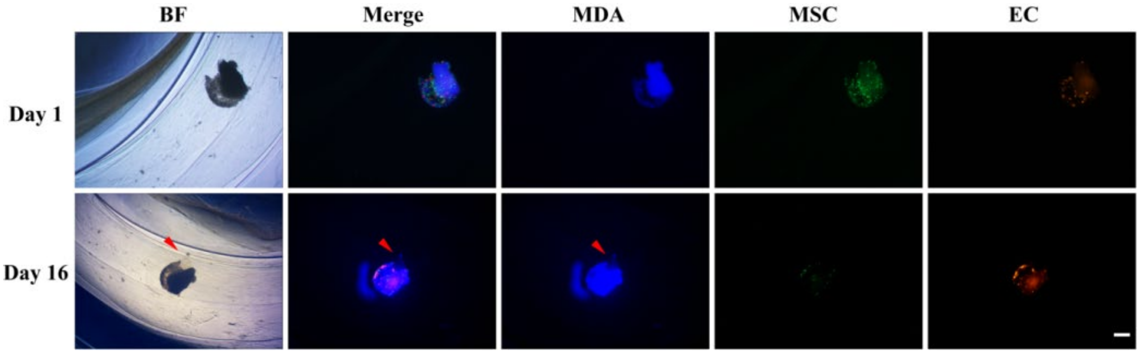

Supplementary Figure 17: MDA STEMs exhibit collective migration in a 3D-Bioprinted model system. Image sequences showing the process of formation of a cell cluster (tumor metastatic unit) from a STEMs and the invasion of the cell cluster (day 16) into the collagen zone, scale bar: 500μm. Red arrows point to the cell cluster.

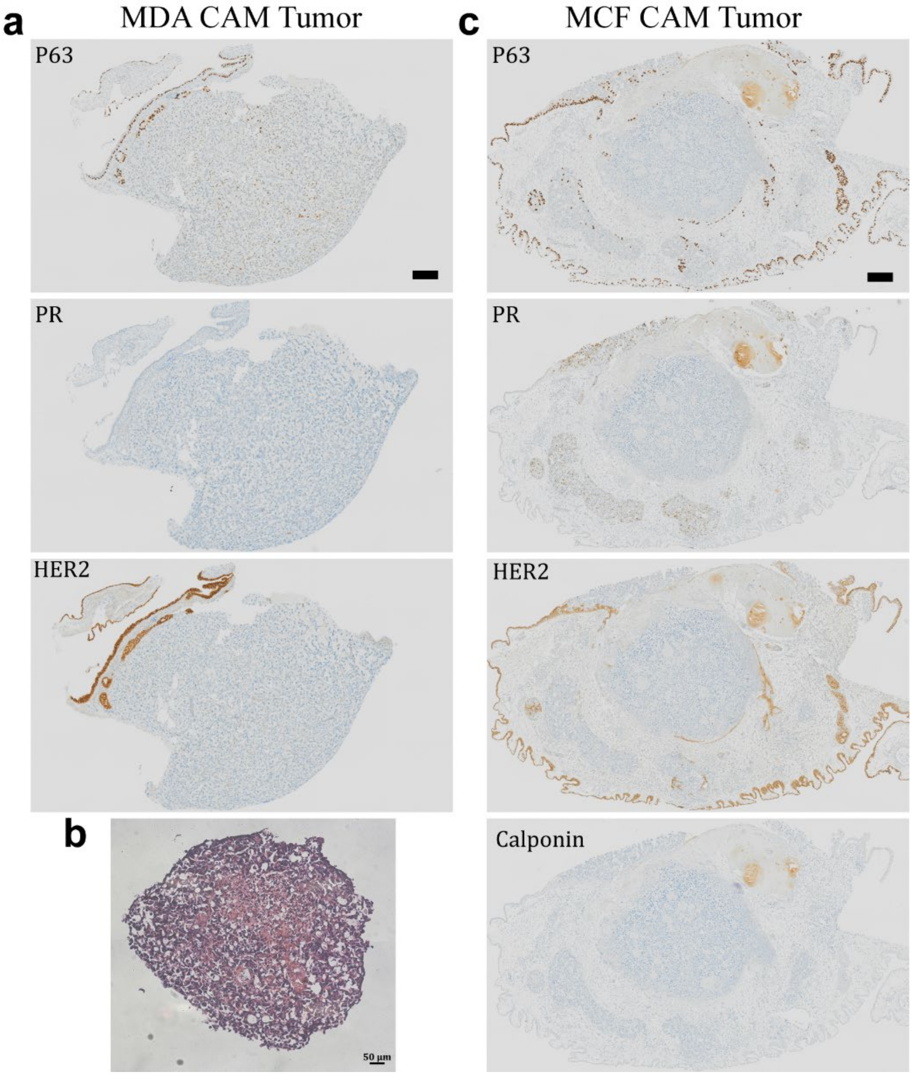

Supplementary Figure 18: Morphological characteristics of MDA and MCF CAM tumors: (a) Immunohistochemical staining of MDA CAM tumor. Cancer cells were negative for human p63, PR, and Her2 (ERBB2). Nuclear p63 positivity in some BCC is indicative of a more aggressive or “metaplastic” subtype of BCC. Note the positive internal control for Her2 on the chicken chorionic membrane. Scale bar: 100 µm. **(b)** H&E staining reveals MCF STEMs did not show the cribriform organization of epithelial cells. **(c)** Immunohistochemical staining of MCF CAM tumor. Cancer cells were positive for PR, but negative for human p63 and Her2 (ERBB2), confirming that the CAM tumors were formed from human BC cells and more specifically from luminal MCF-7 cells. The absence of myoepithelial cells (p63 -ve, Calponin -ve) is indicative of an invasive BC phenotype. Scale bar: 100 µm.

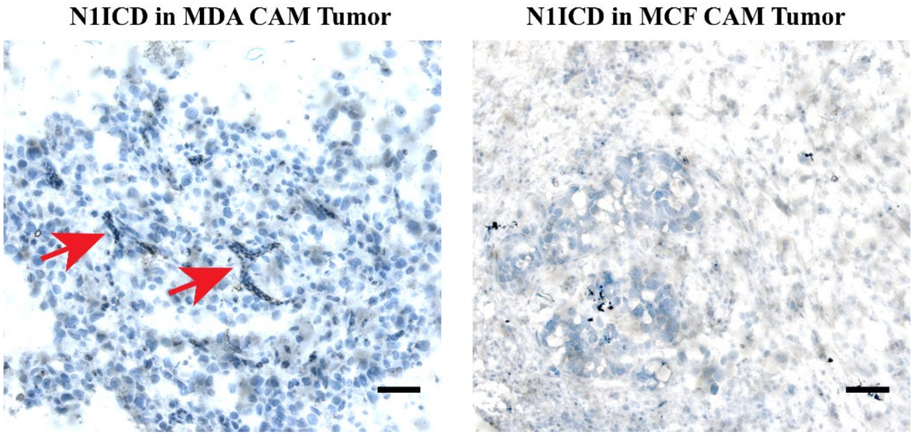

Supplementary Figure 19: Immunohistochemical staining for N1ICD (activated Notch-1) in MDA and MCF CAM tumors. The blood vessels staining (brown color) of Notch-1 is clearly evident in MDA CAM tumor, while MCF CAM tumor does not exhibit such staining, scale bar: 100 μm. Red arrows indicate activated Notch-1 blood vessels.

a

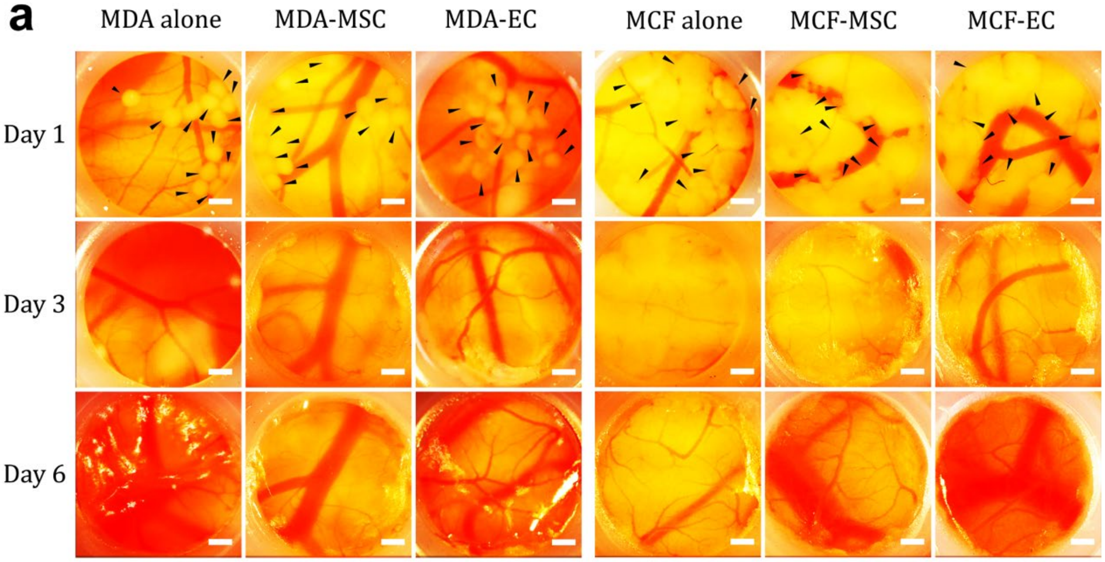

b

CAM tumors formation on the CAM

Treatment	MDA alone	MDA-MSC	MDA-EC	MCF-7alone	MCF-MSC	MCF-EC
Treated embryos	17	16	18	18	19	18
Tumors on CAM	0	0	0	0	0	0
% of CAM tumors	0	0	0	0	0	0

Supplementary Figure 20: CAMs were inoculated with spheroids of MDA alone, MCF alone, and STEMs with the absence of MSC or EC, and their fate followed over 6 days. (a) Exemplar optical micrographs showing none of the spheroids were capable of forming CAM tumors. Day 1: Black arrows point to spheroids on CAM, Day 3: all the spheroids had dissociated. Scale bar: 500 μm. **(b)** Table of percentage of CAM tumors formation by spheroids of MDA alone, MDA-MSC, MDA-EC, MCF-7 alone, MCF-MSC and MCF-EC on CAM.

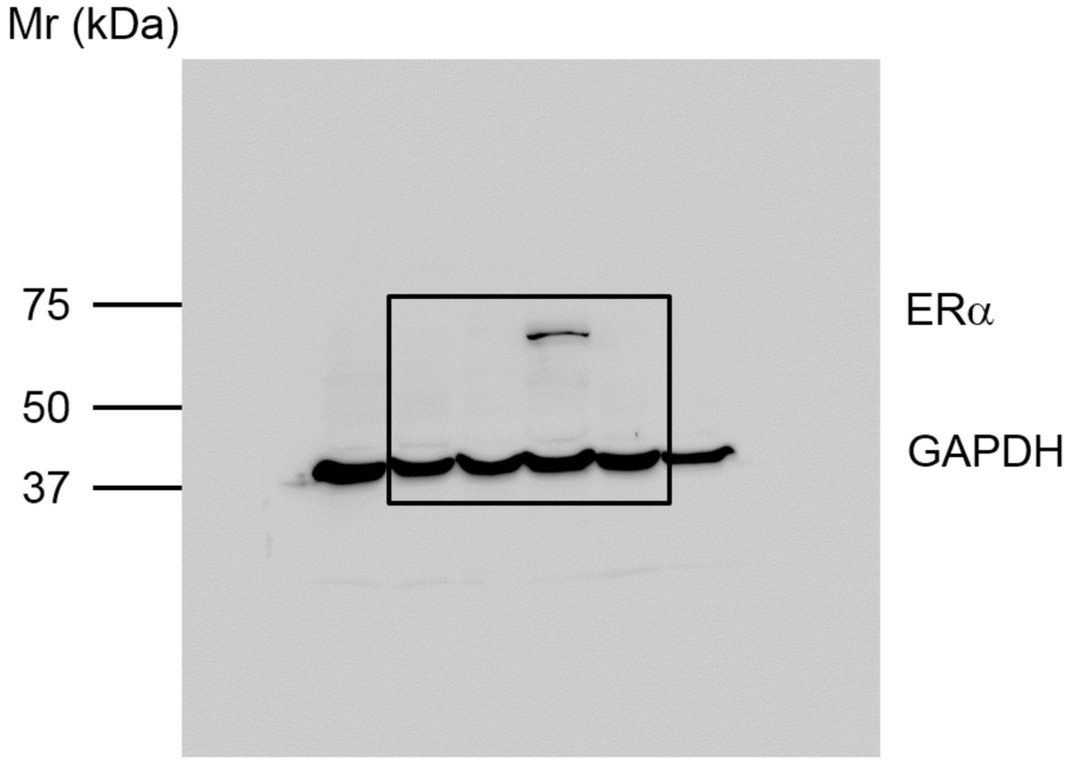

Supplementary Figure 21: Unprocessed image of the one shown in Figure 4a.

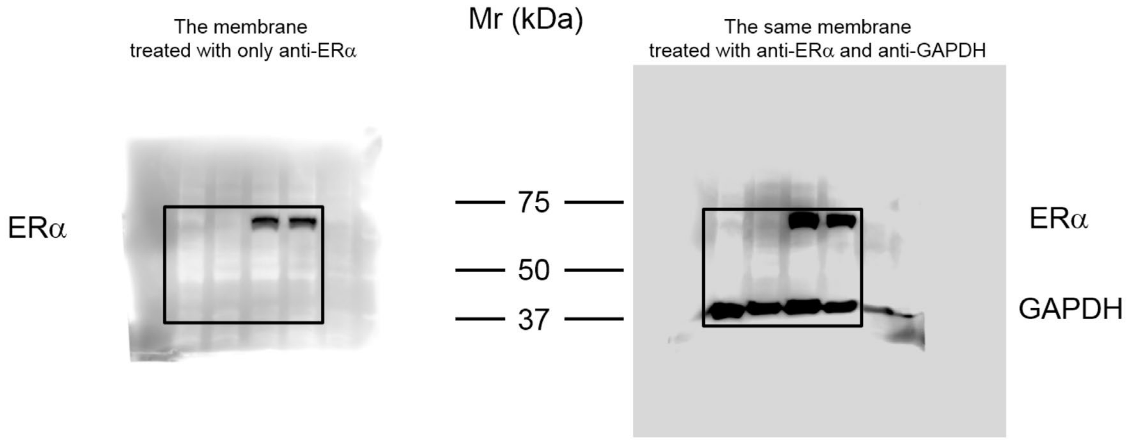

Supplementary Figure 22: Unprocessed image of the one shown in supplementary Figure 13a.

Supplementary Table 1: The clinically relevant panel of markers for human BC

Marker	Structure	Clinical relevance
H&E	Nucleus/extracellular matrix	the gold standard for pathology to detect cancer[1]
ERα	Human estrogen receptor alpha	Breast epithelial cell marker, breast cancer phenotype marker [2]
PR	Human progesterone receptor	Breast epithelial cell marker, breast cancer phenotype marker [3]
Her2	Human epidermal growth factor receptor 2	Breast cancer phenotype marker, is a human growth factor receptor that is overexpressed in approximately 15% of invasive breast cancer and associated with aggressive behavior [4]
CK18	A filamentous protein expressed by epithelial cells and part of the intracytoplasmic cytoskeleton	Primarily expressed by epithelial cells of liver, kidney, breast, prostate, and gastrointestinal tract [5]
CK22	Cytokeratin cocktail	A pan epithelial marker [6]
Calponin	Calcium-binding protein	Lack of calponin-positive structures is indicative of invasive breast cancer [7]
CD-34	The cluster of differentiation marker -34	A marker for human endothelial cells and used to identify vascular structures [8]
p63	Myoepithelial marker	Positivity is suggestive of a more aggressive or “metaplastic” subtype of breast cancer [7, 9]
CD106 (VCAM-1)	Cluster of differentiation marker-106	A protein that functions as a cell adhesion molecule. It is expressed on both large and small blood vessels only after the endothelial cells have been stimulated by cytokines [10]**.** Also, a putative mesenchymal phenotype marker [11]
Ki-67	Nuclear protein	Associated with cellular proliferation, and expressed during all active phases of the cell cycle (G1, S, G2, and mitosis), but is absent in resting (quiescent) cells (G0) [12]
Hu-Mito	Human mitochondrial	A marker for human cells

1. Fischer, A.H., Jacobson, K.A., Rose, J. & Zeller, R. Hematoxylin and eosin staining of tissue and cell sections. *Cold spring harbor protocols* **2008**, pdb. prot4986 (2008).
2. Hua, H., Zhang, H., Kong, Q. & Jiang, Y. Mechanisms for estrogen receptor expression in human cancer. *Exp Hematol Oncol* **7**, 24 (2018).
3. Mohsin, S.K. *et al.* Progesterone receptor by immunohistochemistry and clinical outcome in breast cancer: a validation study. *Mod Pathol* **17**, 1545-1554 (2004).
4. Schillaci, R. *et al.* Clinical relevance of ErbB-2/HER2 nuclear expression in breast cancer. *BMC Cancer* **12**, 74 (2012).
5. Menz, A. *et al.* Diagnostic and prognostic impact of cytokeratin 18 expression in human tumors: a tissue microarray study on 11,952 tumors. *Mol Med* **27**, 16 (2021).
6. Schiavo, G. *et al.* Deregulated HOX genes in ameloblastomas are located in physical contiguity to keratin genes. *J Cell Biochem* **112**, 3206-3215 (2011).
7. Russell, T.D. *et al.* Myoepithelial cell differentiation markers in ductal carcinoma in situ progression. *Am J Pathol* **185**, 3076-3089 (2015).
8. Fina, L. *et al.* Expression of the CD34 gene in vascular endothelial cells. *Blood* **75**, 2417-2426 (1990).
9. Koker, M.M. & Kleer, C.G. p63 expression in breast cancer: a highly sensitive and specific marker of metaplastic carcinoma. *Am J Surg Pathol* **28**, 1506-1512 (2004).
10. Kong, D.H., Kim, Y.K., Kim, M.R., Jang, J.H. & Lee, S. Emerging Roles of Vascular Cell Adhesion Molecule-1 (VCAM-1) in Immunological Disorders and Cancer. *Int J Mol Sci* **19** (2018).
11. Yang, Z.X. *et al.* CD106 identifies a subpopulation of mesenchymal stem cells with unique immunomodulatory properties. *PLoS One* **8**, e59354 (2013).
12. Inwald, E.C. *et al.* Ki-67 is a prognostic parameter in breast cancer patients: results of a large population-based cohort of a cancer registry. *Breast Cancer Research and Treatment* **139**, 539-552 (2013).
