# Supplementary material for: Mesenchymal-endothelial nexus in breast cancer spheroids induces vasculogenesis and local invasion in a CAM model
Source: Commun Biol. 2022 Nov 27;5:1303. doi: 10.1038/s42003-022-04236-5 (PMC9701219; doi:10.1038/s42003-022-04236-5)
Supplement: Supplementary file 2 — Description of Additional Supplementary Data [file 42003_2022_4236_MOESM2_ESM.docx]

**Description of Additional Supplementary Files**

**File name:** Supplementary Data 1

**Description:** the source data for Figure 1c, 1e,2c,2d,3d and Supplementary Figure 2c,5a,5b,5c,5d,5e,9a,14a,15a

**File name:** Supplementary Movie1

**Description:** Confocal z-stack showing organization of EC into vascular structures in MDA STEMs.

**File name:** Supplementary Movie2

**Description:** Confocal z-stack showing lack of vascular structure in MCF STEMs

**File name:** Supplementary Movie3

**Description:** Confocal z-stack showing the absence of EC organization into vascular structures in MDA-EC spheroid

**File name:** Supplementary Movie4

**Description:** Confocal z-stack showing the effect of introduction of MDA in MCF STEMs on EC organization

**File name:** Supplementary Movie5

**Description:** Confocal z-stack showing the organization of EC into vascular structures in MiaPaCa-2 STEMs

**File name:** Supplementary Movie6

**Description:** Confocal z-stack showing the organization of EC into vascular structures in PANC STEMs

**File name:** Supplementary Movie7

**Description:** Confocal z-stack showing the absence of organization of EC into large vascular structures within MCF-10A STEMs
