# Supplementary material for: Mesenchymal-endothelial nexus in breast cancer spheroids induces vasculogenesis and local invasion in a CAM model
Source: Commun Biol. 2022 Nov 27;5:1303. doi: 10.1038/s42003-022-04236-5 (PMC9701219; doi:10.1038/s42003-022-04236-5)
Supplement: Supplementary file 11 — Reporting summary [file 42003_2022_4236_MOESM11_ESM.pdf]

nature portfolio

Corresponding author(s): V. Prasad Shastri

Last updated by author(s): Oct 27, 2022

# Reporting Summary

Nature Portfolio wishes to improve the reproducibility of the work that we publish. This form provides structure for consistency and transparency in reporting. For further information on Nature Portfolio policies, see our Editorial Policies and the Editorial Policy Checklist.

## Statistics

For all statistical analyses, confirm that the following items are present in the figure legend, table legend, main text, or Methods section.

| n/a | Confirmed | |
|---|---|---|
| ☐ | ☒ | The exact sample size (*n*) for each experimental group/condition, given as a discrete number and unit of measurement |
| ☐ | ☒ | A statement on whether measurements were taken from distinct samples or whether the same sample was measured repeatedly |
| ☐ | ☒ | The statistical test(s) used AND whether they are one- or two-sided<br>*Only common tests should be described solely by name; describe more complex techniques in the Methods section.* |
| ☒ | ☐ | A description of all covariates tested |
| ☒ | ☐ | A description of any assumptions or corrections, such as tests of normality and adjustment for multiple comparisons |
| ☐ | ☒ | A full description of the statistical parameters including central tendency (e.g. means) or other basic estimates (e.g. regression coefficient) AND variation (e.g. standard deviation) or associated estimates of uncertainty (e.g. confidence intervals) |
| ☐ | ☒ | For null hypothesis testing, the test statistic (e.g. $F$, $t$, $r$) with confidence intervals, effect sizes, degrees of freedom and $P$ value noted<br>*Give P values as exact values whenever suitable.* |
| ☒ | ☐ | For Bayesian analysis, information on the choice of priors and Markov chain Monte Carlo settings |
| ☒ | ☐ | For hierarchical and complex designs, identification of the appropriate level for tests and full reporting of outcomes |
| ☐ | ☒ | Estimates of effect sizes (e.g. Cohen's *d*, Pearson's *r*), indicating how they were calculated |

*Our web collection on statistics for biologists contains articles on many of the points above.*

## Software and code

Policy information about availability of computer code

| | |
|---|---|
| Data collection | Pearson correlation coefficient were determined using ZEN blue 2.6 software (Carl Zeiss, Germany). Migration and invasion data were collected by ImageJ (version 1.52a) |
| Data analysis | For all statistical analyses, Microsoft Excel (2016) and OriginPro 2022 (OriginLab Corporation, MA, USA) were used. |

For manuscripts utilizing custom algorithms or software that are central to the research but not yet described in published literature, software must be made available to editors and reviewers. We strongly encourage code deposition in a community repository (e.g. GitHub). See the Nature Portfolio guidelines for submitting code & software for further information.

## Data

Policy information about availability of data

All manuscripts must include a data availability statement. This statement should provide the following information, where applicable:

- Accession codes, unique identifiers, or web links for publicly available datasets
- A description of any restrictions on data availability
- For clinical datasets or third party data, please ensure that the statement adheres to our policy

All data necessary to support our conclusions are included in the main manuscript and the supplementary information files. All source data are available within the Supplementary Data. Uncropped gels of Figure 4a and supplementary Figure 13a shows in Supplementary Figure 21 and 22. All other data are available from the corresponding author on reasonable request.

# Field-specific reporting

Please select the one below that is the best fit for your research. If you are not sure, read the appropriate sections before making your selection.

☒ Life sciences ☐ Behavioural & social sciences ☐ Ecological, evolutionary & environmental sciences

For a reference copy of the document with all sections, see nature.com/documents/nr-reporting-summary-flat.pdf

# Life sciences study design

All studies must disclose on these points even when the disclosure is negative.

| | |
|---|---|
| Sample size | We used at least triplicates for each sample and condition because this is standard for typical experiments. |
| Data exclusions | No data was excluded in the manuscript unless otherwise specified. |
| Replication | All experimental results were reliably reproduced. |
| Randomization | Random five fields per membrane of transwell were imaged and cells were counted using ImageJ. Fertilized chicken eggs of CAM assay were randomly chosed. |
| Blinding | Blinding was not required in this study. |

# Reporting for specific materials, systems and methods

We require information from authors about some types of materials, experimental systems and methods used in many studies. Here, indicate whether each material, system or method listed is relevant to your study. If you are not sure if a list item applies to your research, read the appropriate section before selecting a response.

## Materials & experimental systems

| n/a | Involved in the study | |
|---|---|---|
| ☐ | ☒ | Antibodies |
| ☐ | ☒ | Eukaryotic cell lines |
| ☒ | ☐ | Palaeontology and archaeology |
| ☒ | ☐ | Animals and other organisms |
| ☒ | ☐ | Human research participants |
| ☒ | ☐ | Clinical data |
| ☒ | ☐ | Dual use research of concern |

## Methods

| n/a | Involved in the study | |
|---|---|---|
| ☒ | ☐ | ChIP-seq |
| ☒ | ☐ | Flow cytometry |
| ☒ | ☐ | MRI-based neuroimaging |

## Antibodies

| | |
|---|---|
| Antibodies used | Activated Notch1 antibody (D3B8; #4147; Cell Signaling Technology), CK18 (Clone B23.1, Ventana), CK22 (Clone MM-1012-02, Immuno Bio), Calponin (Clone 760-4376, Ventana), CD34 (Clone QBEnd/10, Ventana), Ki-67 (Clone IR626, Dako), ERalpha (Clone SP1, Ventana), PR (Clone 1E2, Ventana), Her2 (Clone 4B5, Ventana), p63 (Clone 4A4, Ventana), human mitochondria (MAB1273, Millipore), CD106 (MA5-16429, Thermo Fisher), ER alpha antibody (Santa Cruz Biotechnology, D-12,1:500), human Podocalyxin anti-rabbit (1:200, HPA002110; Sigma). |
| Validation | Notch1 antibody was validated by 376 of product citations; References: Nitin Roper, et. al.Notch signaling and efficacy of PD-1/PD-L1 blockade in relapsed small cell lung cancer, Nat Commun, 2021; Renata Ferrarotto, et. al.Proteogenomic Analysis of Salivary Adenoid Cystic Carcinomas Defines Molecular Subtypes and Identifies Therapeutic Targets, Clin Cancer Res,2021;<br>ERalpha antibody (Santa Cruz Biotechnology, D-12,1:500) was validated by (1.Mason, B.H., et al. 1983. Progesterone and estrogen receptors as prognostic variables in breast cancer. Cancer Res. 43: 2985-2990. 2.Evans, R.M. 1988. The steroid and thyroid hormone receptor superfamily. Science 240: 889-895. 3.Danielian, P.S., et al. 1992. Identification of a conserved region required for hormone dependent transcriptional activation by steroid hormone receptors. EMBO J. 11: 1025-1033. 4.Kliewer, S.A., et al. 1992. Retinoid X receptor interacts with nuclear receptors in retinoic acid, thyroid hormone and vitamin D3signaling. Nature 355: 446-449.)<br>Human mitochondria antibody was validated in immunohistochemistry applications (Ko, H.R., et al. (2018). Stem Cell Res Ther.;9(1):326; MacAskill, M.G., et al. (2018). Mol Ther. 26(7):1669-1684);<br>CD106 antibody was validated by references (Immunological and ultrastructural characterization of endothelial cell cultures differentiated from human cord blood derived endothelial progenitor cells; Effect of maternal anti-HPA-1a antibodies and polyclonal IVIG on the activation status of vascular endothelial cells;Irradiation of mechanically-injured human arterial endothelial cells leads to increased gene expression and secretion of inflammatory and growth promoting cytokines);<br>CK18;CK22;Calponin;CD34;Ki-67 ;ERalpha;PR;Her2 and p63 antibodies were validated by Institute for Medical Genetics and Pathology (University Hospital Basel, Basel 4031, Switzerland);<br>Podocalyxin antibody was validated by our previous publication(Polysaccharide hydrogels with tunable stiffness and provasculogenic properties via α-helix to β-sheet switch in secondary structure). |

## Eukaryotic cell lines

Policy information about cell lines

| | |
|---|---|
| Cell line source(s) | The human breast cancer cell lines (MDA-MB-231 and MCF-7), the human pancreatic cancer cell lines (Mia Paca-2 and PNAC-1), MCF-10A, and HEK293 cells were provided by the toolbox of BIOSS (Centre for Biological Signalling Studies, University of Freiburg), Human pulmonary microvascular endothelial cells (HPMEC) were purchased from PromoCell (Heidelberg, Germany), Human marrow-derived mesenchymal stem cells (MSCs) were provided by Dr. Andrea Barbero and were obtained from patients under consent per the regulations of the local ethical committee (University Hospital Basel; Ref No: 78/07). BT549 cells were kindly provided by Prof. Dr. Andreas Fischer and were authenticated using Multiplex Cell Authentication by Multiplexion (Heidelberg, Germany). |
| Authentication | The human breast cancer cell lines (MDA-MB-231 and MCF-7), the human pancreatic cancer cell lines (Mia Paca-2 and PNAC-1), MCF-10A, and HEK293 cells were genotyped and verified by Labor für DNA Analytik (Freiburg, Germany). Human pulmonary microvascular endothelial cells (HPMEC) and Human marrow-derived mesenchymal stem cells (MSCs) were authenticated based on their morphology by microscopy and verified by Promocell or University Hospital Basel. BT549 cells were authenticated using Multiplex Cell Authentication by Multiplexion (Heidelberg, Germany). |
| Mycoplasma contamination | All types of cells were tested by Eurofins Scientific and were negative for mycoplasma. |
| Commonly misidentified lines (See ICLAC register) | No misidentified cell lines were used. |
